# Commensal Leucothoidae (Crustacea, Amphipoda) of the Ryukyu Archipelago, Japan. Part III: coral rubble-dwellers

**DOI:** 10.3897/zookeys.173.2498

**Published:** 2012-03-02

**Authors:** Kristine N. White, James Davis Reimer

**Affiliations:** 1 Rising Star Program, Trans-disciplinary Organization for Subtropical Island Studies (TRO-SIS), University of the Ryukyus, 1 Senbaru, Nishihara, Okinawa, Japan 903-0213

**Keywords:** Leucothoidae, Ryukyus, Okinawa, Japan, new species, commensal, *Anamixis sentan*, *Leucothoe akaisen*, *Leucothoe akuma*, *Leucothoe chiisainame*, *Leucothoe enko*, *Leucothoe kebukai*, *Paranamixis misakiensis*

## Abstract

Commensal leucothoid amphipods have been collected from coral rubble samples throughout the Ryukyu Archipelago, Japan. Seven new species are described in two generawith valuable location data. A new locality is presented for *Paranamixis misakiensis* Thomas, 1997. An identification key to all described Leucothoidae of the Ryukyu Archipelago is provided.

## Introduction

The Leucothoidae are a family of marine gammaridean amphipods that can be found inhabiting sessile invertebrate hosts worldwide. Including the species described here, the family contains 163 species in five genera and can be divided into two clades, the anamixid clade and the leucothoid clade after a wide revision of the group made by White (2011). Table 1 lists the diagnostic characters for each clade. Leucothoids are typically found as endocommensal associates of sponges, ascidians, and bivalve mollusks, where they utilize the feeding current produced by their hosts to feed (White 2011; White and Reimer 2012a).

**Table 1.** Diagnostic morphological characters of anamixid and leucothoid clades, based on White, 2011.

Character	Anamixid clade	Leucothoid clade
Mouthparts	Reduced	Well developed
Coxa 1	Reduced	Similar in size to coxa 2
Gnathopod 2	Elongate	Generally shortened
Sexual dimorphism	Extreme	Minimal to moderate
Juvenile morphology	Dissimilar to adult males	Similar to adult males

Collection of amphipods throughout the Ryukyu Archipelago has increased the number of leucothoid species known from Japan from seven to 32, with 25 of these occurring in the Ryukyu Archipelago (White and Reimer 2012a, b). Seven new species are described, six from the genus *Leucothoe* and one from the genus *Anamixis*. The currently recognized biogeographic boundaries for terrestrial organisms within the Ryukyu Archipelago (see Figure 1 in White and Reimer 2012a) due to the past connection of the island chain to the Eurasian continent by land bridges (Kizaki and Oshiro 1977, 1980; Hikida and Ota 1997; Ota 1998; Ota et al. 2004) do not appear to apply to these marine species (White and Reimer 2012a, b).

## Methods

Specimens were collected via snorkeling and SCUBA at 47 locations throughout the Ryukyu Archipelago: Ishigaki–jima Island (4 locations), Iriomote–jima Island (4), Okinawa–jima Island (21), Yoron–to Island (2), Okinoerabu–jima Island (2), Tokunoshima Island (4), Amami–oshima Island (6), and Yakushima Island (4) (see map in White and Reimer 2012a). Detailed station data is available in Supplementary Table 1 in White and Reimer (2012a).

One 12 liter bucket was filled with colorful, sponge-filled coral rubble (Figure 16) at each location. The bucket contents were then elutriated and sieved on location using both saltwater and formalin washes. Samples were sorted immediately. Amphipods were preserved in 2% seawater buffered formalin for morphological analysis and 99% ethanol for molecular studies.

Specimens used for morphological analyses were transferred to glycerin, dissected, mounted on slides, and illustrated using a Nikon®Y-IDT drawing tube attached to a Nikon® Eclipse 50I compound microscope. Pencil drawings were scanned and digitally inked in Adobe® Illustrator using a Wacom® Tablet, following the methods of Coleman (2003).

Descriptions are of males unless noted with sexually dimorphic characters described in a separate section. Terminology used in descriptions follows White and Thomas (2009) with ‘proximal margin’ of the carpus and dactylus referring to the margins closing on the propodus. Setae nomenclature follows Oshel and Steele (1988) where possible without having SEM images for the specimens described here. All setae are simple, unless noted.

Type material is deposited in the University of the Ryukyus Museum (Fujukan), with the prefix RUMF for museum numbers. Additional material has been deposited in the National Museum of Nature and Science in Tokyo, with the prefix NSMT for museum numbers.

Scale bars in figures represent 0.1 mm unless noted.

Figure legend: Hd, head; Mx, maxilla; Md, mandible; Mdp, mandibular palp; Xpd, maxilliped; LL, lower lip; UL, upper lip; G, gnathopod; P, pereopod; T, telson; U, uropod; L, left; R, right; l, lateral; m, medial; p, paratype; +, enlarged.

## Taxonomy

### Anamixis

Stebbing, 1897

#### Diagnosis.

 (Anamorph males) Antennae long. Maxilliped inner plates apically fused, sometimes with small cleft; outer plates lacking inner lobes. Coxa 1 greatly reduced, remainder of gnathopod 1 present, occasionally vestigial.

### Anamixis sentan sp. n.

urn:lsid:zoobank.org:act:690636D9-C67C-4454-B51B-21353E88E312

http://species-id.net/wiki/Anamixis_sentan

Figures 1 2

#### Type material.

 Holotype, anamorph male, 2.2 mm RUMF-ZC-1837, Sunabe Seawall, Okinawa–jima Island, Okinawa, spur and groove reef (26°19'25"N, 127°44'43"E), among coral rubble, 3–12 m, K.N. White and N.S. White, col., 17 February 2011 (KNWOkinawa31E). Paratype leucomorph female, 2.1 mm, RUMF-ZC-1838, Sunabe Seawall, Okinawa–jima Island, Okinawa, spur and groove reef (26°19'25"N, 127°44'43"E), among coral rubble, 6–8 m, K.N. White and N.S. White, col., 5 October 2010 (KNWOkinawa12D). Paratype leucomorph male, 1.2 mm, RUMF-ZC-1839, same station data as holotype.

#### Type locality.

 Sunabe Seawall, Okinawa–jima Island, Okinawa, Japan (26°19'25"N, 127°44'43"E).

#### Additional material examined.

 1 anamorph male, RUMF-ZC-1840, KNWOkinawa24A; 1 anamorph male, NSMT-Cr 21985, KNWTokuno4F; 2 leucomorphs, RUMF-ZC-1841, KNWOkinawa24A; 1 leucomorph, RUMF-ZC-1842, KNWTokuno4F; 1 leucomorph, NSMT-Cr 21986, KNWTokuno4F; 1 leucomorph, RUMF-ZC-1843, KNWOkinawa13C; 4 leucomorphs, NSMT-Cr 21987, KNWOkinawa16H; 1 leucomorph, RUMF-ZC-1844, KNWOkino1B; 1 leucomorph, NSMT-Cr 21988, KNWOkino1B; 3 leucomorphs, RUMF-ZC-1845, KNWOkino2C; 2 leucomorphs, NSMT-Cr 21989, KNWOkino1A; 2 leucomorphs, NSMT-Cr 21990, KNWYoron1B.

#### Diagnosis.

 Anamorph male head anterior margin truncate, anterodistal margin quadrate with cusp; ventral cephalic keel anteroventral margin quadrate with anteriorly projecting cusp. Maxilliped inner plates serrate with small cleft. Gnathopod 2 coxa distal margin with mid-distal projection and posterodistal cusp; propodus palm with 1 large and 1 small triangular projection; dactylus proximal margin setose with indentation. Leucomorph male or female head anterior margin truncate with anterodistal indentation; ventral cephalic keel anterior margin produced and distally excavate.

#### Description (Anamorph male).

 Head. Anterior margin truncate, anterodistal margin quadrate with cusp; ventral cephalic keel anterior margin transverse, anteroventral margin quadrate with anteriorly projecting cusp, ventral margin excavate; eyes present with more than 10 ommatidia, round. Antenna 1 0.4 × body length, flagellum 9–articulate, peduncle article 1 width less than 2 × article 2, accessory flagellum 1–articulate. Antenna 2 0.3 × body length, shorter than antenna 1, flagellum 4–articulate. Mouthparts reduced. Maxilliped inner plates bare; outer plate inner margin smooth, reaching 0.1 × palp article 1, bare; palp 4–articulate, article 4 elongate, distally acute.

Pereon. Pereonite 1 with lateral locking ridge. Coxae 1–4 relative widths 1.0: 1.4: 1.5: 1.3. Gnathopod 1 coxa reduced, anterodistal margin produced, subtriangular, bi-cuspidate, distal margin excavate, posterior margin oblique, facial setae absent; basis linear, anterior and posterior margins bare; ischium bare; carpal lobe curved distally, distal length 7.7 × width, proximal margin smooth, distal margin bare, terminal ornamentation absent; propodus curved, palm serrate with several short proximal setae; dactylus absent. Gnathopod 2 coxa longer than broad, subequal in size with coxa 3, smooth, with tiny marginal setae, anterior margin expanded, anterodistally rounded, distal margin rounded, with mid-distal projection and posterodistal cusp, posterior margin straight, facial setae absent; basis distally expanded, anterior margin with 1 small distal tubercle and 2 short setae, posterior margin bare, ischium bare; carpus 0.9 × propodus length, straight, distally tapered, anterior margin smooth; propodus with 1 mediofacial setal row above midline, reaching 0.3 × propodus length, without submarginal setae, posterior margin smooth, palm linear, with 1 large and 1 small triangular projection distally; dactylus straight, reaching 0.3 × propodus length, proximal margin setose with indentation, anterior margin distally obtuse. Pereopod 3 coxa length 1.3 × width, anterodistal corner overriding distal face of coxa 2, not extending below it, smooth, bare, anterior margin expanded, distal margin oblique with posterodistal cusp, posterior margin rounded, facial setae absent. Pereopod 4 coxa smooth, bare, anterior margin produced, distal margin oblique with mid-distal cusp, posterior margin tapered, facial setae absent. Pereopods 5–7 coxae, facial setae absent; bases width length ratios 1: 1.4, 1: 1.4, 1: 1.3, posterior margins smooth, bare.

Pleon. Epimera 1–3 bare; epimeron 3 posteroventral corner quadrate, produced. Uropods 1–3 relative lengths 1.0: 0.8: 1.1. Uropod 1 peduncle 0.9 × inner ramus length, outer ramus length 0.4 × inner ramus length; inner ramus with 3 robust setae, outer ramus with 1 robust seta. Uropod 2 peduncle 0.8 × inner ramus length, outer ramus 0.4 × inner ramus length; inner ramus with 2 robust setae, outer ramus with 1 robust seta. Uropod 3 peduncle 1.1 × inner ramus length, outer ramus 0.8 × inner ramus length; inner ramus with 3 robust setae, outer ramus with 2 robust setae. Telson 1.1 × longer than wide, apex rounded.

**Figure 1.**
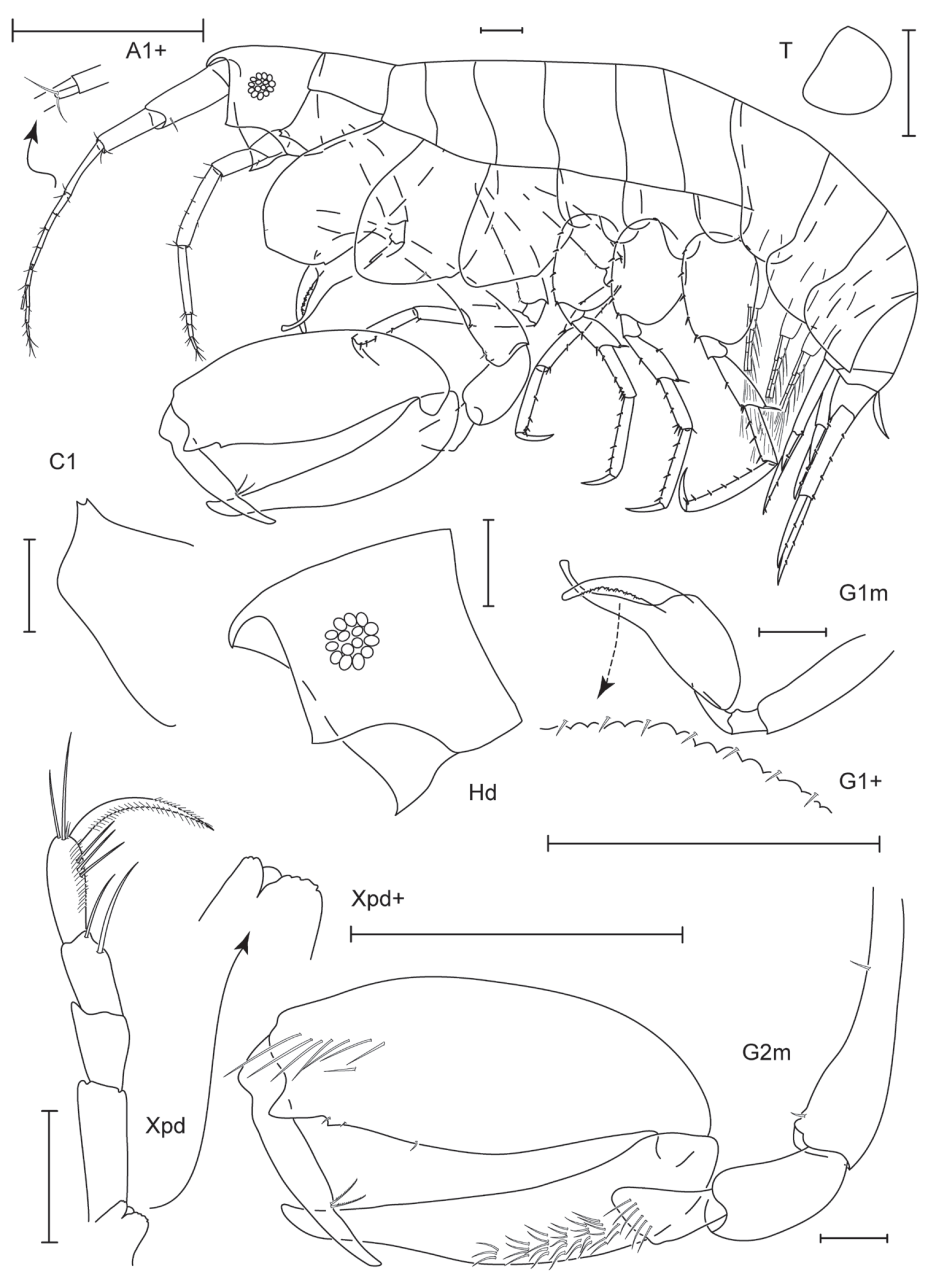
*Anamixis sentan* sp. n., holotype anamorph male, 2.2 mm, RUMF-ZC-1837.

**Figure 2.**
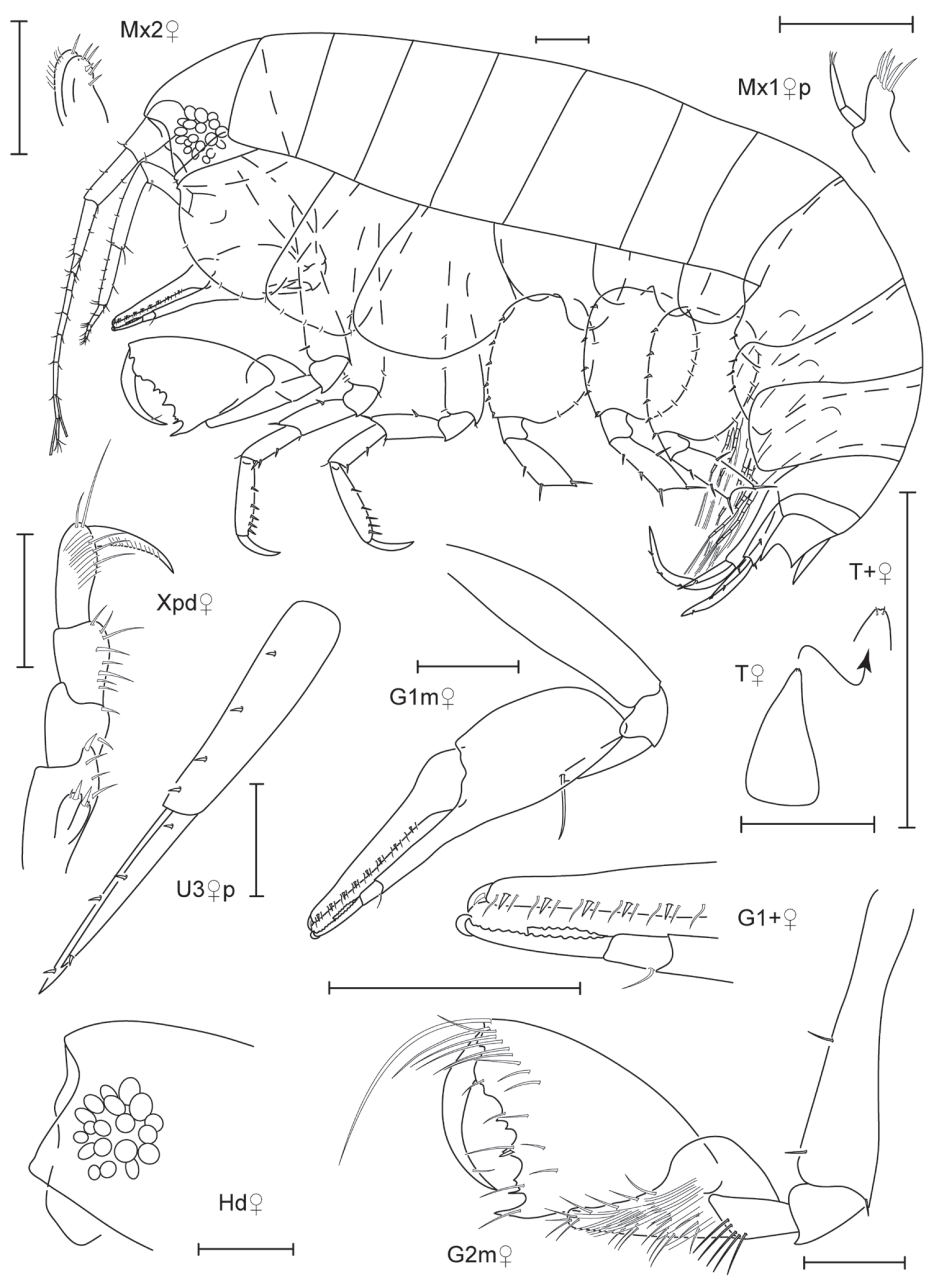
*Anamixis sentan* sp. n., paratype leucomorph female, 2.1 mm, RUMF-ZC-1838.

#### Leucomorph (juvenile and sexually dimorphic characters).

 Head. Anterior margin truncate, with small anterodistal indentation, anterodistal margin subquadrate, distal margin straight. Ventral cephalic keel anterior margin produced and distally excavate, anteroventral margin rounded, ventral margin projected downward. Antenna 1 0.3 × body length, flagellum 8–articulate, peduncle article 1 width less than 2 × article 2, accessory flagellum absent. Antenna 2 0.2 × body length, shorter than antenna 1, flagellum 3–articulate. Mandibles, upper lip, and lower lip lost in dissection. Maxilla 1 palp 2–articulate with 3 distal setae; outer plate with 5 distal robust setae. Maxilla 2 inner plate with 4 slender setae and 1 robust distal seta; outer plate with 1 distal seta and 8 marginal setae. Maxilliped inner plates fused, distal margin with v-shaped indentation, with large and small robust setae; outer plate inner margin smooth, reaching 0.3 × palp article 1, with simple marginal setae.

Pereon. Coxae 1–4 relative widths 1.0: 2.1: 1.5: 2.2. Gnathopod 1 coxa smooth, with tiny marginal setae, anterior margin oblique, distal margin rounded with cusp, posterior margin oblique, facial setae absent; basis distally expanded, anterior and posterior margins bare; ischium bare; carpus linear, with unclear articulation at distal end, distal length 11.8 × width, proximal margin smooth, distal margin with 1 distal seta, with 2 terminal serrate blades, apically rounded; propodus straight, palm smooth with 8 sets of 2 slender and 1 robust proximal setae; dactylus smooth, reaching less than 0.1 × propodus length. Gnathopod 2 coxa longer than broad, larger than coxa 3, smooth, with tiny marginal setae, anterior margin produced, anterodistally rounded, distal margin rounded, with anterodistal cusp, posterior margin straight, facial setae absent; basis distally expanded, anterior margin with 2 setae, posterior margin with 1 distal seta; ischium bare; carpus 0.3 × propodus length, straight, distally rounded, anterior margin smooth; propodus with 1 mediofacial setal row above midline, reaching 0.3 × propodus length, with 1 row of submarginal setae, posterior margin smooth, palm subtriangular with 7 small and large pointed projections; dactylus curved, reaching 0.4 × propodus length, proximal margin smooth, setose, anterior margin distally acute. Pereopod 3 coxa length 1.5 × width, anterodistal corner overriding distal face of coxa 2 and extending below it, smooth, with tiny marginal setae, all margins straight, corners rounded, facial setae absent. Pereopod 4 coxa smooth, setose, anterior margin straight, distal margin rounded, posterior margin excavate. Pereopods 5–7 coxae facial setae absent; bases length width ratios 1: 1.2, 1: 1.1, 1: 1.2.

Pleon. Epimera 1–2 with ventral setae, epimeron 3 bare, posteroventral corner subquadrate. Uropods 1–3 relative lengths 1.0: 0.7: 1.4. Uropod 1 peduncle 0.7 × inner ramus length, outer ramus 0.4 × inner ramus length; inner ramus with 3 robust setae, outer ramus with 1 robust seta. Uropod 2 peduncle 0.7 × inner ramus length, outer ramus 0.6 × inner ramus length; inner ramus with 1 robust seta, outer ramus with 2 robust setae. Uropod 3 peduncle 1.1 × inner ramus length, outer ramus 0.9 × inner ramus length ; inner ramus with 2 robust setae, outer ramus with 3 robust setae. Telson 1.9 × longer than width, apex with weak indentation.

#### Etymology.

 After the Japanese word ‘sentan’, meaning ‘pointy end’ and referring to the angular head and ventral cephalic keel (pronounced sen-tahn).

#### Ecology.

 Host unknown, presumably living in sponges in coral rubble.

#### Relationships.

*Anamixis sentan* sp. n. fits into the *Anamixis bazimut* Thomas, 1997, *Anamixis kateluensis* Thomas, 1997, and *Anamixis moana* Thomas, 1997group introduced by Thomas (1997). These species share several characters, including a truncate anterior head margin with the ventral cephalic keel extending below the distal head margin and similar shape and morphology of gnathopod 2. *Anamixis sentan* sp. n. shares the cleft maxilliped inner plates with *Anamixis bazimut* and *Anamixis kateluensis*. *Anamixis sentan* sp. n. is distinct from all other *Anamixis* species in having serrate maxilliped inner plates in the anamorph; gnathopod 1 coxa anterodistal margin bi-cuspidate, distal margin excavate, and propodus palm smooth with 8 sets of 2 slender and 1 robust proximal setae in the leucomorph.

#### Remarks.

Anamorph males of *Anamixis sentan* sp. n. are white in color with 1 robust magenta-pink stripe on each pereonite segment (Figure 15A). Leucomorph males and females are translucent with very faint pink stripes along pereonite edges; some specimens appear to be translucent pink in color (Figure 15B). This species appears to be endemic to the central Ryukyu Islands. The anamorph and leucomorph of this species have not been collected together directly from their host, yet more than one year of collecting has revealed only two anamorph morphologies and two leucomorph morphologies. One anamorph and leucomorph morphology belong to *Paranamixis thomasi* White and Reimer, 2012a. The other anamorph and leucomorph morphology are described here as *Anamixis sentan*, although there is a very slight chance that this leucomorphbelongs to a different anamorph counterpart that has not been collected.

#### Distribution.

 East China Sea: Ishigaki–jima Island, Okinawa–jima Island (both Okinawa), Yoron–to Island, Okinoerabu–jima Island, Tokunoshima Island (all Kagoshima), Japan.

### Leucothoe

Leach, 1814

#### Generic diagnosis.

 Eyes, if present, generally well developed with 10 or more ommatidia. Mandibles lacking molars, palp three articulate; right lacinia mobilis smaller than left. Maxilliped inner plates fused, palp 4–articulate; outer plates not reaching apex of palp article 1. Coxa 1–4 relatively equal in widths. Pereopods 5–7 bases generally expanded. Minimal to no sexual dimorphism.

### Leucothoe akaisen sp. n.

urn:lsid:zoobank.org:act:76556C59-E6CF-4080-B622-8F6DDCFD6721

http://species-id.net/wiki/Leucothoe_akaisen

Figures 3 4

#### Type material.

Holotype male, 2.8 mm RUMF-ZC-1854, Sunabe Seawall, Okinawa–jima Island, Okinawa, spur and groove reef (26°19'25"N, 127°44'43"E), among coral rubble, 6–9 m, K.N. White and N.S. White, col., 16 October 2010 (KNWOkinawa12E). Paratype female, 3.4 mm, RUMF-ZC-1855, Yona, Kunigami, Okinawa–jima Island, Okinawa, reef wall (26°45'56"N, 128°11'46"E), among coral rubble, 9 m, K.N. White and N.S. White, col., 23 October 2010 (KNWOkinawa15F).

#### Type locality.

 Sunabe Seawall, Okinawa–jima Island, Okinawa, Japan (26°19'25"N, 127°44'43"E).

#### Additional material examined.

 3 specimens, NSMT-Cr 21995, KNWOkinawa12E; 1 specimen, RUMF-ZC-1856, KNWOkinawa15F; 1 specimen, RUMF-ZC-1857, KNWOkinawa44H; 1 specimen, NSMT-Cr 21996, KNWTokuno4E; 1 specimen, RUMF-ZC-1858, KNWIshigaki4H; 2 specimens, NSMT-Cr 21997, KNWIshigaki2F; 1 specimen, RUMF-ZC-1859, KNWIriomote3C; 4 specimens, RUMF-ZC-1860, KNWYaku3O; 2 specimens, NSMT-Cr 21998, KNWYaku1D; 2 specimens, NSMT-Cr 21999, KNWYaku5J.

#### Diagnosis (male).

 Head anterior margin rounded. Maxilla 1 palp 1–articulate. Gnathopod 1 coxa anterodistal margin produced, subquadrate, with 1 long medial facial seta; dactylus reaching 0.3 × propodus length. Gnathopod 2 propodus mediofacial setal row displaced past midline. Pereopods 5–7 bases broadly expanded. Epimeron 1 with tuft of anteroventral setae. Telson apex rounded.

#### Description (male).

 Head. Anterior margin rounded, anterodistal margin evenly rounded, distal margin straight; ventral cephalic keel anterior margin transverse, anteroventral margin rounded, ventral margin straight; eyes present with more than 10 ommatidia, round. Antenna 1 0.3 × body length, flagellum 5–articulate, peduncle article 1 width less than 2 × article 2, accessory flagellum absent. Antenna 2 0.2 × body length, shorter than antenna 1, flagellum 3–articulate. Mandibular palp ratio of articles 1–3 1.0: 2.6: 1.0, article 2 with 5 distal setae, article 3 with two distal setae, incisors weakly dentate; left mandible with 11 raker spines, lacinia mobilis large, weakly toothed; right mandible with 8 raker spines, lacinia mobilis small. Upper lip asymmetrically lobate, anterior margin setose. Lower lip inner lobes fused, setose; outer lobes with large gape, anterior margins setose. Maxilla 1 palp 1–articulate, with 4 distal setae; outer plate with 6 distal robust setae and 2 distal setae. Maxilla 2 inner plate with 9 distal and 1 proximal setae; outer plate with 4 distal and 11 marginal setae. Maxilliped inner plates distal margin rounded with v-shaped indentation, with large and small robust setae; outer plate inner margin smooth, reaching 0.4 × palp article 1, with simple marginal setae, facial setae present; palp article 4 subequal in length with article 3, distally acute.

Pereon. Coxae 1–4 relative widths 1.0: 1.2: 0.7: 1.6. Gnathopod 1 coxa smooth, with tiny marginal setae, anterodistal margin produced, subquadrate, distal margin straight, posterior margin excavate, facial seta present; basis slightly inflated, anterior margin with 1 short and 2 medium setae, posterior margin bare; ischium bare; carpus linear, distal length 10.7 × width, proximal margin smooth, distal margin with 2 setae; propodus straight, palm dentate with 2 large and several small distal setae; dactylus smooth, reaching 0.3 × propodus length. Gnathopod 2 coxa longer than broad, slightly larger than coxa 3, smooth, with tiny marginal setae, anterior margin straight, anterodistally rounded, distal and posterior margins straight, facial setae absent; basis slightly inflated, anterior margin with 4 setae, posterior margin with 1 posterodistal seta; ischium with posterodistal seta; carpus 0.4 × propodus length, curved, distally tapered, anterior margin smooth; propodus with 1 mediofacial setal row displaced past midline, reaching 0.8 × propodus length, with 1 row of submarginal setae, posterior margin smooth, palm convex, with 3 small rounded tubercles; dactylus curved, reaching 0.5 × propodus length, proximal margin smooth, bare, anterior margin distally acute. Pereopod 3 coxa length 1.9 × width, anterodistal corner overriding distal face of coxa 2 and extending below it, smooth, with tiny marginal setae, anterior margin straight, distal margin oblique, posterior margin straight, facial setae absent. Pereopod 4 coxa smooth, with tiny marginal setae, anterior margin tapered, distal margin produced, posterior margin excavate, facial setae absent. Pereopods 5–6 coxae, facial setae present, pereopod 7 coxa, facial setae absent. Pereopods 5–7 bases width length ratios 1: 1.2, 1: 1.3, 1: 1.0, posterior margins smooth, setose.

Pleon. Epimeron 1 with anteroventral tuft of setae, epimeron 2 with ventral setae, epimeron 3 bare, posteroventral corner subquadrate, produced. Uropods 1–3 relative lengths 1.0: 0.7: 1.0. Uropod 1 peduncle subequal in length with inner ramus, outer ramus 0.9 × inner ramus length; inner and outer rami each with 2 robust setae. Uropod 2 peduncle subequal in length with inner ramus, outer ramus 0.8 × inner ramus length; inner and outer rami each with 2 robust setae. Uropod 3 peduncle 0.9 × inner ramus length, outer ramus subequal in length with inner ramus; inner ramus with 3 robust setae, outer ramus with 1 robust seta. Telson 2.2 × longer than wide, apex rounded.

**Figure 3.**
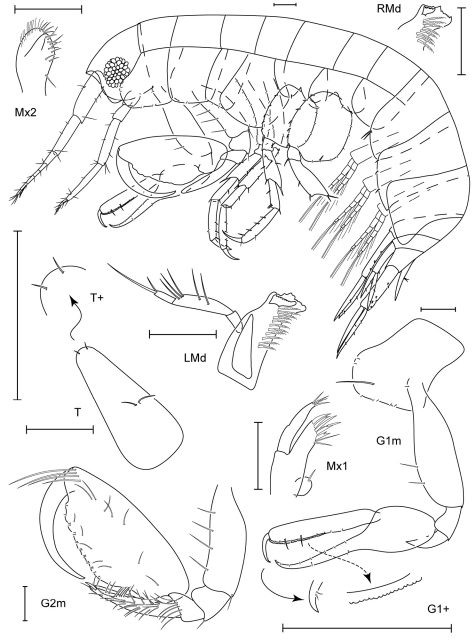
*Leucothoe akaisen* sp. n., holotype male, 2.8 mm, RUMF-ZC-1854.

**Figure 4.**
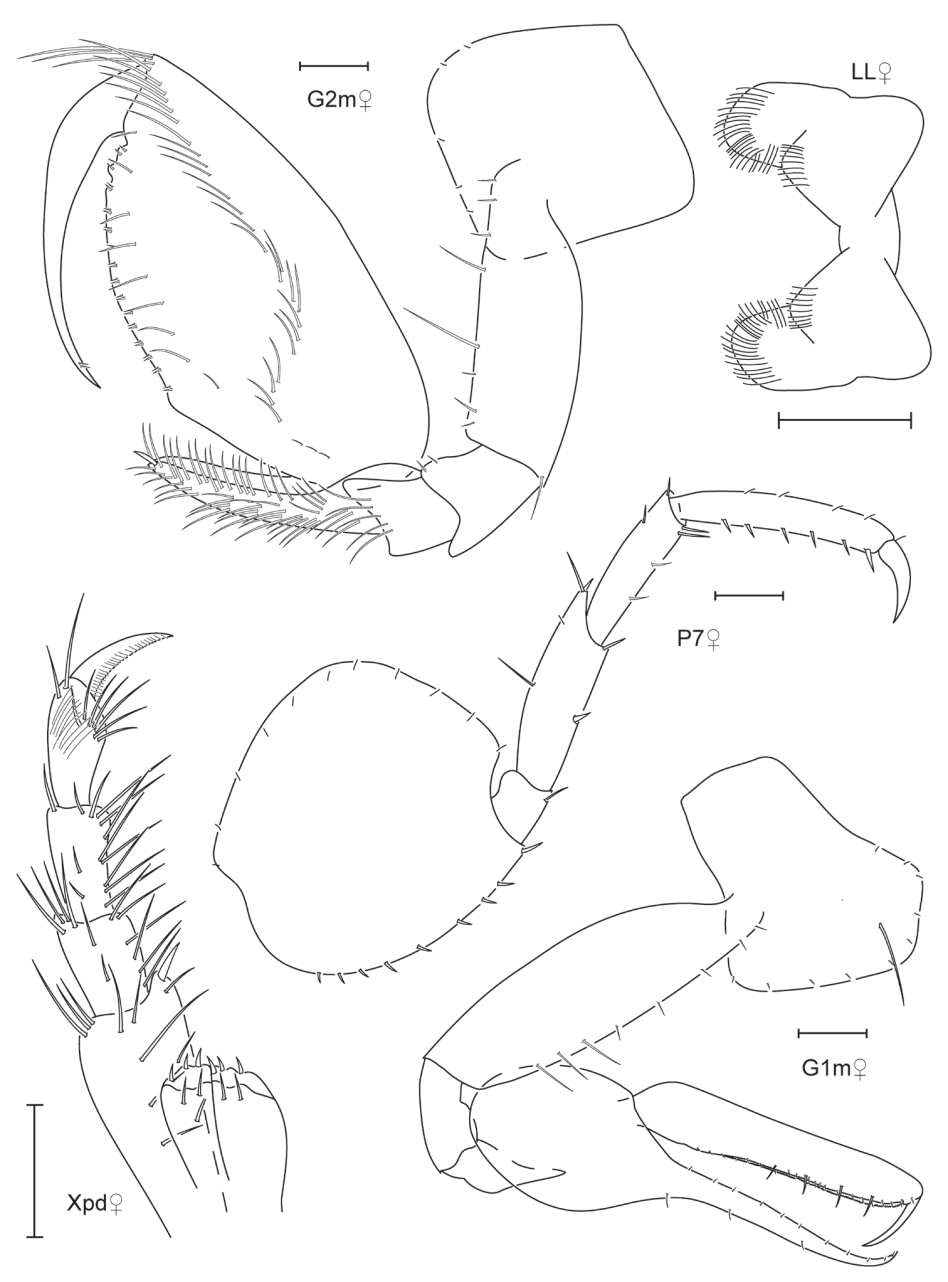
*Leucothoe akaisen* sp. n., paratype female, 3.4 mm, RUMF-ZC-1855.

#### Female (sexually dimorphic characters).

Gnathopod 1 basis anterior margin with 5 short and 3 medium setae; carpus distal margin with 3 setae; propodus palm with 4 large and several small distal setae. Gnathopod 2 basis anterior margin with 8 setae; ischium with 2 short anterodistal setae; propodus mediofacial setal row extended proximally with 3 very short setae.

#### Etymology.

 After the Japanese words ‘akai’, meaning ‘red’ and ‘sen’, meaning ‘line’ and referring to the red stripes along the antenna 1 peduncle (pronounced ah-ky-sen).

#### Ecology.

 Host unknown, presumably in sponges in coral rubble.

#### Relationships.

*Leucothoe akaisen* sp. n. is similar to *Leucothoe trulla* White & Reimer, 2012a, *Leucothoe commensalis* Haswell, 1879, *Leucothoe wuriti* Thomas & Klebba, 2007, *Leucothoe epidemos* White & Thomas, 2009, and *Leucothoe thula* White & Thomas, 2009. The members of this “*Leucothoe commensalis* group” share a rounded head, long gnathopod 1 dactylus, a displaced gnathopod 2 propodus mediofacial setal row, and wide pereopod 5–7 bases. *Leucothoe akaisen* sp. n. shares several additional characters with *Leucothoe thula*, including a maxilla 1 palp with an unclear articulation, a tapered gnathopod 2 carpus, and the gnathopod 2 propodus palm with small tubercles, all of which differ from the other species in this group. *Leucothoe akaisen* sp. n. is unique in having a longer antenna 1 flagellum, gnathopods 1 and 2 dactylus with proximal setae, and a rounded telson apex.

#### Remarks.

*Leucothoe akaisen* sp. n. is very unique in coloration. This species has deep red stripes along the peduncle of antenna 1, a large yellow eye, a yellow “saddleback” pattern, and a deep red dot on coxa 4 (Figure 15D). This species is endemic to the Ryukyus Archipelago, having been collected throughout the island chain. The color pattern of this species resembles that of an undescribed species collected in Guam from polychaete worm tubes (Thomas, pers.com.).

#### Distribution.

 East China Sea: Ishigaki–jima Island, Iriomote–jima Island, Okinawa–jima Island (both Okinawa), Tokunoshima Island, Yakushima Island (both Kagoshima), Japan.

### Leucothoe akuma sp. n.

urn:lsid:zoobank.org:act:92D41396-9D89-4BE4-8851-B08DD693101C

http://species-id.net/wiki/Leucothoe_akuma

Figures 5 6

#### Type material.

Holotype male, 2.9 mm RUMF-ZC-1861, Toguchi Beach, Okinawa–jima Island, Okinawa, patch reef (26°21'47"N, 127°44'12"E), among coral rubble, 1–2 m, K.N. White and N.S. White, col., 20 October 2010 (KNWOkinawa14I). Paratype female, 2.5 mm, RUMF-ZC-1862, Toguchi Beach, Okinawa–jima Island, Okinawa, patch reef (26°21'47"N, 127°44'12"E), among coral rubble, 1–2 m, K.N. White and N.S. White, col., 9 September 2011 (KNWOkinawa63A).

#### Type locality.

 Toguchi Beach, Okinawa–jima Island, Okinawa, Japan (26°21'47"N, 127°44'12"E).

#### Additional material examined.

 1 specimen, RUMF-ZC-1863, KNWOkinawa39N; 1 specimen, RUMF-ZC-1864, KNWOkinawa41D; 2 specimens, NSMT-Cr 22000, KNWOkinawa63A; 2 specimens, NSMT-Cr 22001, KNWOkinawa72A.

#### Diagnosis (male).

 Head anterior margin rounded; ventral cephalic keel anterior margin excavate, anteroventral margin quadrate, projected anteriorly. Antenna 1 accessory flagellum 1–articulate. Gnathopod 1 dactylus reaching 0.3 × propodus length. Gnathopod 2 carpus distally truncate; propodus with 2 mediofacial setal rows, secondary row reaching to insertion of dactylus. Pereopods 5–6 coxae with facial setae; bases broadly expanded.

#### Description (male).

 Head. Anterior margin rounded, anterodistal margin evenly rounded, distal margin rounded; ventral cephalic keel anterior margin excavate, anteroventral margin quadrate, projected anteriorly, ventral margin convex; eyes present with more than 10 ommatidia, round. Antenna 1 0.3 × body length, flagellum 10–articulate, peduncle article 1 width less than 2 × article 2, accessory flagellum 1–articulate. Antenna 2 0.3 × body length, subequal in length with antenna 1, flagellum 7–articulate. Mandibular palp ratio of articles 1–3 1.0: 2.6: 1.8, article 2 with 8 distal setae, article 3 with one distal seta; incisors strongly dentate; left mandible with 7 raker spines, lacinia mobilis large, weakly toothed; right mandible with 7 raker spines, lacinia mobilis small, weakly toothed. Upper lip asymmetrically lobate, anterior margin setose. Lower lip inner lobes fused, setose; outer lobes with small gape, anterior margins setose. Maxilla 1 palp 2-articulate with 3 distal setae; outer plate with 5 distal robust setae. Maxilla 2 inner plate with 7 distal setae; outer plate with 7 slender distal setae, 4 slender and 4 robust marginal setae. Maxilliped inner plates distal margin with v-shaped indentation, with short simple robust setae; outer plate inner margin smooth, reaching 0.3 × palp article 1, with simple marginal setae, facial setae absent; palp article 4 subequal in length with article 3, distally acute.

Pereon. Coxae 1–4 relative widths 1.0: 1.1: 0.7: 1.3. Gnathopod 1 coxa smooth, with tiny marginal setae, anterodistal margin produced, subquadrate, distal margin straight, posterior margin excavate, facial setae absent; basis distally expanded, anterior margin with 3 short setae, posterior margin bare; ischium bare; carpus linear, distal length 12.9 × width, proximal margin smooth, distal margin with 3 setae; propodus straight, palm dentate with 5 large and several small distal setae; dactylus smooth, reaching 0.3 × propodus length. Gnathopod 2 coxa broader than long, subequal in size with coxa 3, smooth, with tiny marginal setae, anterior margin straight, anterodistally rounded, distal and posterior margins straight, facial setae absent; basis distally expanded, anterior margin with 7 short and long setae, posterior margin bare except for 1 small posterodistal seta; ischium with distal and posterodistal setae; carpus 0.4 × propodus length, curved, distally truncate, anterior margin dentate; propodus with 2 mediofacial setal rows, primary row above midline, reaching 0.7 × propodus length, secondary row reaching to insertion of dactylus, with 1 row of submarginal setae, posterior margin smooth, palm convex with 2 large distal tubercles; dactylus curved, proximal margin smooth, setose, anterior margin distally acute, reaching 0.6 × propodus length. Pereopod 3 coxa length 1.4 × width, anterodistal corner overriding distal face of coxa 2 and extending below it, smooth, with tiny marginal setae, anterior margin straight, distal margin convex, posterior margin straight, facial setae absent. Pereopod 4 smooth, with tiny marginal setae, anterior margin straight, distal margin rounded, posterior margin tapered, facial setae absent. Pereopods 5–7 coxae facial setae present; bases width length ratios 1: 1.3, 1: 1.2, 1: 1.1, posterior margins smooth. Pereopod 5 basis posterior margin bare, pereopods 6–7 bases posterior margins setose.

Pleon. Epimera 1–3 with ventral setae; epimeron 3 posteroventral corner rounded. Uropods 1–3 relative lengths 1.0: 0.7: 1.0. Uropod 1 peduncle 0.9 × inner ramus length, outer ramus length 0.8 × inner ramus length; inner ramus with 5 robust setae, outer ramus with 4 robust setae. Uropod 2 peduncle 0.8 × inner ramus length, outer ramus 0.7 × inner ramus length; inner ramus with 4 robust setae, outer ramus with 3 robust setae. Uropod 3 peduncle and outer ramus subequal in length with inner ramus; inner ramus with 5 robust setae, outer ramus with 3 robust setae. Telson 2.4 × longer than wide, apex tridentate.

**Figure 5.**
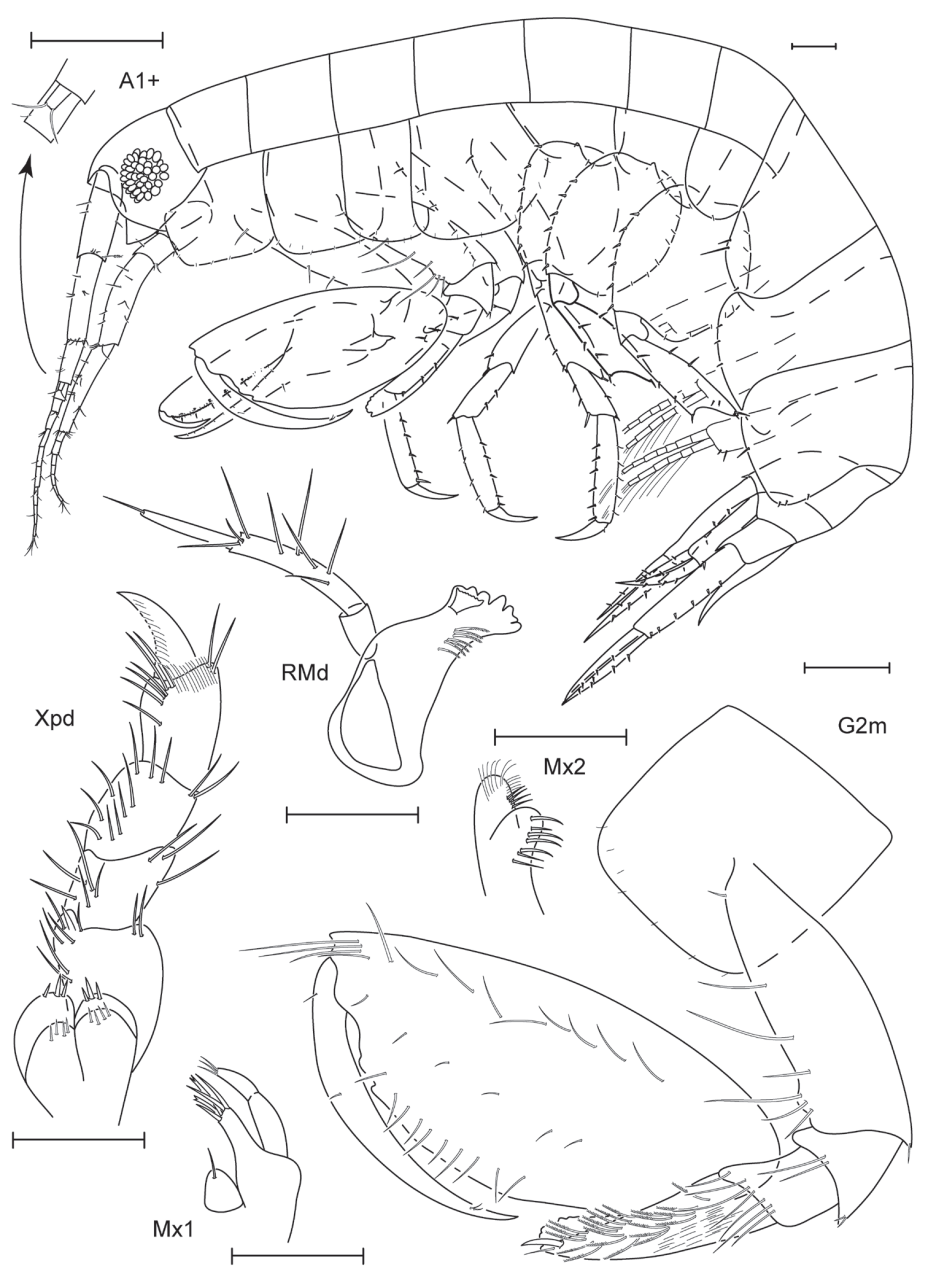
*Leucothoe akuma* sp. n., holotype male, 2.9 mm, RUMF-ZC-1861.

**Figure 6.**
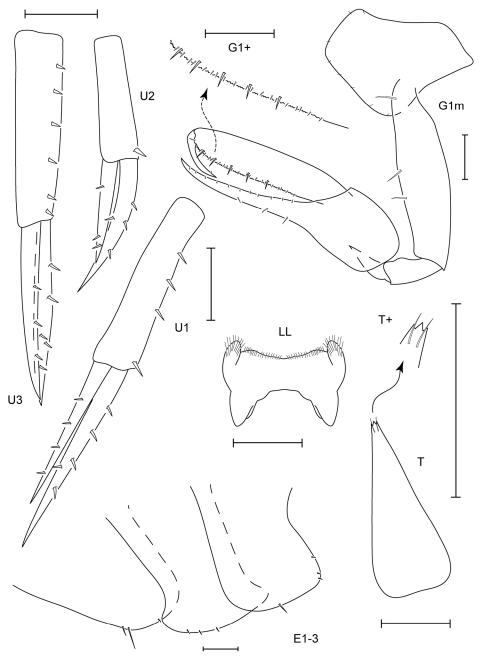
*Leucothoe akuma* sp. n., holotype male, 2.9 mm, RUMF-ZC-1861.

#### Female (sexually dimorphic characters).

 Gnathopod 1 carpus posterodistal margin with 2 setae; propodus palm with 6 large and several small distal setae. Gnathopod 2 basis anterior margin with 10 long setae, distal margin with 2 long setae.

#### Etymology.

 After the Japanese word ‘akuma’, meaning ‘devil’ and referring to the type locality, also known as ‘Devil’s Cove’ (pronounced ah-koo-ma).

#### Ecology.

 Host unknown, presumably in sponges in coral rubble.

#### Relationships.

*Leucothoe akuma* sp. n. is similar to *Leucothoe togatta* White & Reimer, 2012b, *Leucothoe hashi* White & Reimer, 2012b, and *Leucothoe amamiensis* White & Reimer, 2012a in having a rounded anterodistal head margin and a ventral cephalic keel with an excavate anterior margin and a produced, quadrate anteroventral margin. *Leucothoe akuma* sp. n. differs from *Leucothoe hashi* in the overall shape of gnathopod 1 and from the other species in having mandibular palp article 2 with fewer distal setae, lower lip with setose inner lobes, gnathopod 2 carpus longer and distally truncate, and gnathopod 2 propodus medial surface with two rows of submarginal setae. This last unique character is shared only with *Leucothoe enko* sp.n.

#### Remarks.

*Leucothoe akuma* sp. n. is white in color with magenta-pink stripes along pereonite edges (Figure 15H). This species is endemic to the central Ryukyu Islands.

#### Distribution.

 East China Sea: Okinawa–jima Island (Okinawa) and Okinoerabu–jima Island (Kagoshima), Japan.

### Leucothoe chiisainame sp. n.

urn:lsid:zoobank.org:act:968C3205-8DF1-4CF1-B9BF-CDF68C5EA86C

http://species-id.net/wiki/Leucothoe_chiisainame

Figures 7 , 8

#### Type material.

Holotype female, 4.2 mm RUMF-ZC-1874, Kurio, Yakushima Island, Kagoshima, reef wall (30°16'00"N, 130°24'55"E), among coral rubble, 7–10 m, K.N. White and N.S. White, col., 26 May 2011 (KNWYaku1J).

#### Type locality.

 Kurio, Yakushima Island, Kagoshima, Japan (30°16'00"N, 130°24'55"E).

#### Additional material examined.

 No additional material available for examination.

#### Diagnosis (female).

 Head anterior margin excavate; ventral cephalic keel anterior margin excavate, anteroventral margin produced, rounded; eyes small. Antenna 1 accessory flagellum 1–articulate. Right mandible lacinia mobilis with 2 rows of dentition. Maxilla 2 inner plate with 3 rows of 2, 2, and 3 distal setae, with 3 marginal setae. Maxilliped inner plates with wide v-shaped indentation distally. Gnathopod 1 coxa anterodistally serrate; propodus palm with square-shaped dentition. Gnathopod 2 carpus distally truncate. Pereopods 5–6 coxae with facial setae. Pereopods 5–7 bases broadly expanded, posteriorly serrate. Epimeron 1 with 1 anteroventral seta.

#### Description (female).

 Head. Anterior margin excavate, anterodistal margin rounded, distal margin straight; ventral cephalic keel anterior margin excavate, anteroventral margin produced, rounded, ventral margin convex; eyes present with more than 10 ommatidia, round, small. Antenna 1 0.3 × body length, flagellum 7–articulate, peduncle article 1 width less than 2 × article 2, accessory flagellum 1–articulate. Antenna 2 0.2 × body length, shorter than antenna 1, flagellum 4–articulate. Mandibular palp broken in dissection, incisors strongly dentate; left mandible with 9 raker spines, lacinia mobilis large, strongly toothed; right mandible with 11 raker spines, lacinia mobilis small with 2 rows of dentition. Upper lip asymmetrically lobate, anterior margin setose. Lower lip lost in dissection. Maxilla 1 palp 2-articulate with 3 distal setae; outer plate with 5 distal robust setae. Maxilla 2 inner plate with 3 rows of 2, 2, and 3 distal setae, with 3 proximal marginal setae; outer plate with 2 distal robust setae and 13 marginal setae. Maxilliped inner plates with wide v-shaped indentation distally, with short simple robust setae and long slender setae; outer plate inner margin smooth, reaching 0.5 × palp article 1, with simple marginal setae, facial setae present; palp article 4 subequal in length with article 3, distally acute.

Pereon. Coxae 1–4 relative widths 1.0: 1.0: 0.8: 1.4. Gnathopod 1 coxa with tiny marginal setae, anterodistal margin produced, subquadrate, serrate, distal margin straight, posterior margin excavate, facial setae absent; basis distally expanded, anterior margin with 7 short and medium setae, posterior margin bare; ischium with 2 short posterior setae; carpus linear, distal length 10.4 × width, proximal margin dentate, distal margin with 4 setae; propodus straight, palm dentate with 5 distal setae; dactylus smooth, reaching 0.3 × propodus length. Gnathopod 2 coxa broader than long, slightly larger than coxa 3, smooth, with tiny marginal setae, anterior margin expanded, anterodistally rounded, distal and posterior margins rounded, facial setae absent; basis distally expanded, anterior margin with 5 short and medium setae, posterior margin with 2 short setae; ischium with anterodistal, distal, and posterodistal setae; carpus 0.4 × propodus length, curved, distally truncate, anterior margin dentate; propodus with 1 mediofacial setal row displaced past midline, reaching 0.7 × propodus length, with 1 row of submarginal setae, posterior margin smooth, palm convex with 3 major and many small tubercles; dactylus curved, proximal margin smooth, bare, anterior margin distally acute, reaching 0.4 × propodus length. Pereopod 3 coxa length 1.1 × width, anterodistal corner overriding distal face of coxa 2 and extending below it, smooth, with tiny marginal setae, anterior margin straight, distal margin oblique, posterior margin straight, facial setae absent. Pereopod 4 coxa smooth, with tiny marginal setae, anterior margin tapered, distal margin rounded, posterior margin excavate, facial setae absent. Pereopod 5–6 coxae facial setae present, pereopod 7 coxa facial setae absent. Pereopods 5–7 bases width length ratios 1: 1.2, 1: 1.2, 1: 1.2, posterior margins serrate, setose.

Pleon. Epimeron 1 with 1 antero-ventral seta, epimera 2–3 with ventral setae; epimeron 3 posteroventral corner subquadrate, produced. Uropods 1–3 relative lengths 1.0: 0.7: 1.0. Uropod 1 peduncle and outer ramus subequal in length with inner ramus; inner ramus with 3 robust setae, outer ramus with 4 robust setae. Uropod 2 peduncle 0.8 × inner ramus length, outer ramus 0.6 × inner ramus length; inner ramus with 4 robust setae, outer ramus with 3 robust setae. Uropod 3 peduncle and outer ramus 0.9 × inner ramus length; inner ramus with 3 robust setae, outer ramus with 1 robust seta. Telson 2.4 × longer than wide, apex weakly tridentate.

**Figure 7.**
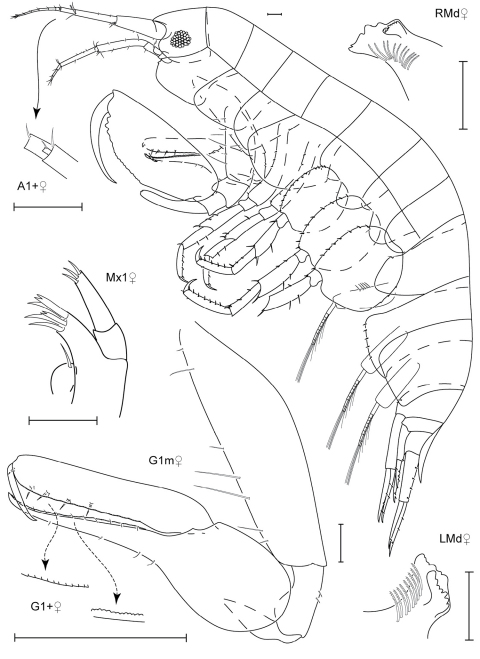
*Leucothoe chiisainame* sp. n., holotype female, 4.2 mm, RUMF-ZC-1874.

**Figure 8.**
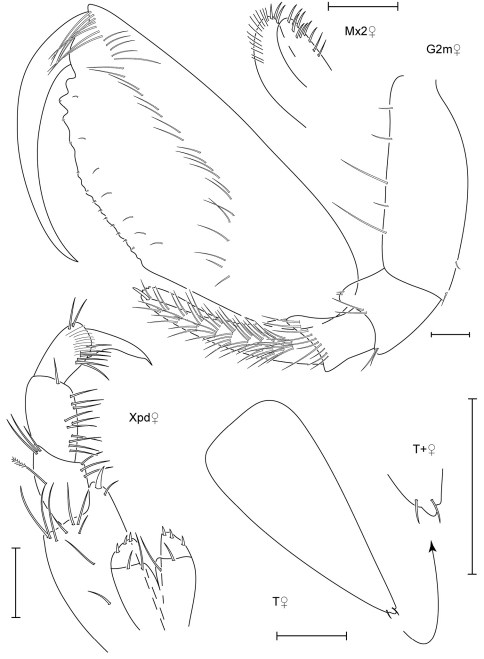
*Leucothoe chiisainame* sp. n., holotype female, 4.2 mm, RUMF-ZC-1874.

#### Male (sexually dimorphic characters).

 Unknown.

#### Etymology.

 After the Japanese words ‘chiisai’, meaning ‘small’ and ‘me’, meaning ‘eye’ and referring to the very small eye (pronounced chee-sy-nah-may).

#### Ecology.

 Host unknown, presumably in sponges in coral rubble.

#### Relationships.

*Leucothoe chiisainame* sp. n. shares a rounded anteroventral head margin, truncate gnathopod 2 carpus, broadly expanded pereopod 5–7 bases, and a tuft of anteroventral setae on epimeron 1 with *Leucothoe alcyone* Imbach, 1967, *Leucothoe occidentalis* (Reid, 1951), *Leucothoe saron* Thomas & Klebba, 2007, and *Leucothoe vulgaris* White & Reimer, 2012a. *Leucothoe chiisainame* sp. n. differs from these species in the following: head anterior margin excavate, eye small; maxilla 2 with rows of apical setae; gnathopod 1 coxa anterodistally serrate; gnathopod 2 dactylus short; pereopods 5–7 bases posteriorly serrate; and epimeron 3 posteroventral corner produced.

#### Remarks.

*Leucothoe chiisainame* sp. n. is white in color with a very small yellow-orange eye (Figure 15F). This species is endemic to Yakushima Island. Although there is only one specimen available for analysis, the authors are confident that this is a new species based on the diagnostic characters.

#### Distribution.

 East China Sea: Yakushima Island, Kagoshima, Japan.

### Leucothoe enko sp. n.

urn:lsid:zoobank.org:act:3CFF4120-9249-4A14-8966-C427EC500A1D

http://species-id.net/wiki/Leucothoe_enko

Figures 9 , 10

#### Type material.

Holotype male, 1.6 mm RUMF-ZC-1850, Omonawa, Tokunoshima Island, Kagoshima, reef wall (27°53'29"N, 128°56'10"E), among coral rubble, 21 m, K.N. White and N.S. White, col., 21 September 2010 (KNWTokuno3B). Paratype female, 1.7 mm, RUMF-ZC-1851, Toguchi Beach, Okinawa–jima Island, Okinawa, patch reef (26°21'47"N, 127°44'12"E), among coral rubble, 1–2 m, K.N. White and N.S. White, col., 20 October 2010 (KNWOkinawa14C).

#### Type locality.

 Omonawa, Tokunoshima Island, Kagoshima, Japan (27°53'29"N, 128°56'10"E).

#### Additional material examined.

 1 specimen, RUMF-ZC-1852, KNWTokuno3B; 4 specimens, NSMT-Cr 21993, KNWOkinawa24C; 1 specimen, RUMF-ZC-1853, KNWOkinawa24C; 1 specimen, NSMT-Cr 21994, KNWOkinawa71B.

#### Diagnosis (male).

 Head anterior margin oblique, produced, distal margin excavate with rounded projection. Maxilla 1 palp 1–articulate, margins constricted. Gnathopod 1 propodus proximally inflated: dactylus reaching 0.1 × propodus length. Gnathopod 2 basis distally expanded, stout; propodus palm concave with 6 large tubercles. Pereopods 5–7 bases narrowly expanded, posterior margins serrate.

#### Description (male).

 Head. Anterior margin oblique, produced, anterodistal margin rounded, distal margin excavate with rounded projection; ventral cephalic keel anterior produced, excavate; anteroventral margin quadrate, ventral margin excavate; eyes present with more than 10 ommatidia, round. Antenna 1 0.3 × body length, flagellum 6–articulate, peduncle article 1 width less than 2 × article 2, accessory flagellum absent. Antenna 2 0.3 × body length, subequal in length with antenna 1, flagellum 4–articulate. Mandibular palp ratio of articles 1–3 1.0: 2.4: 0.6, article 2 with 3 distal setae, article 3 with two distal setae, incisors weakly dentate; left mandible with 5 raker spines, lacinia mobilis large, weakly toothed; right mandible with 5 raker spines, lacinia mobilis small, weakly toothed. Upper lip asymmetrically lobate, anterior margin weakly setose. Lower lip inner lobes fused, setose; outer lobes with moderate gape, anterior margins setose. Maxilla 1 palp 1–articulate, margins constricted with 3 distal slender setae; outer plate with 5 distal robust setae. Maxilla 2 inner plate with 4 distal slender setae outer plate with 3 distal slender setae. Maxilliped inner plates distal margin rounded with v-shaped indentation, with short simple robust setae; outer plate inner margin smooth, reaching 0.2 × palp article 1, with simple marginal setae, facial setae present; palp article 4 subequal in length with article 3, distally acute.

Pereon. Coxae 1–4 relative widths 1.0: 1.1: 0.9:1.0. Gnathopod 1 coxa smooth, bare, anterodistal margin produced, rounded, distal margin straight, posterior margin excavate, facial setae absent; basis distally expanded, anterior and posterior margins bare; ischium bare; carpus linear, distal length 9.1 × width, proximal margin dentate, distal margin with 4 long setae; propodus proximally inflated, palm dentate with 4 distal setae; dactylus smooth, reaching 0.1 × propodus length. Gnathopod 2 coxa broader than long, subequal in size with coxa 3, smooth, bare, anterior margin straight, anterodistally rounded, distal margin rounded, posterior margin straight, facial setae absent; basis distally expanded, stout, anterior margin with 3 setae, posterior margin bare; ischium bare; carpus 0.5 × propodus length, curved, distally tapered, anterior margin smooth; propodus with 1 mediofacial setal row above midline, reaching 0.7 × propodus length, without submarginal setae, posterior margin smooth, palm concave with 6 major and 3 minor tubercles; dactylus curved, proximal margin smooth, bare, anterior margin distally acute, reaching 0.6 × propodus length. Pereopod 3 coxa length 1.2 × width, anterodistal corner overriding distal face of coxa 2, not extending below it, smooth, bare, anterior margin straight, distal margin slightly rounded, posterior margin straight, facial setae absent. Pereopod 4 coxa smooth, bare, anterior margin straight, distal margin rounded, posterior margin straight, facial setae absent. Pereopods 5–7 coxae facial setae absent; bases width length ratios 1: 2.2, 1: 1.9, 1: 1.8, posterior margins serrate, bare.

**Figure 9.**
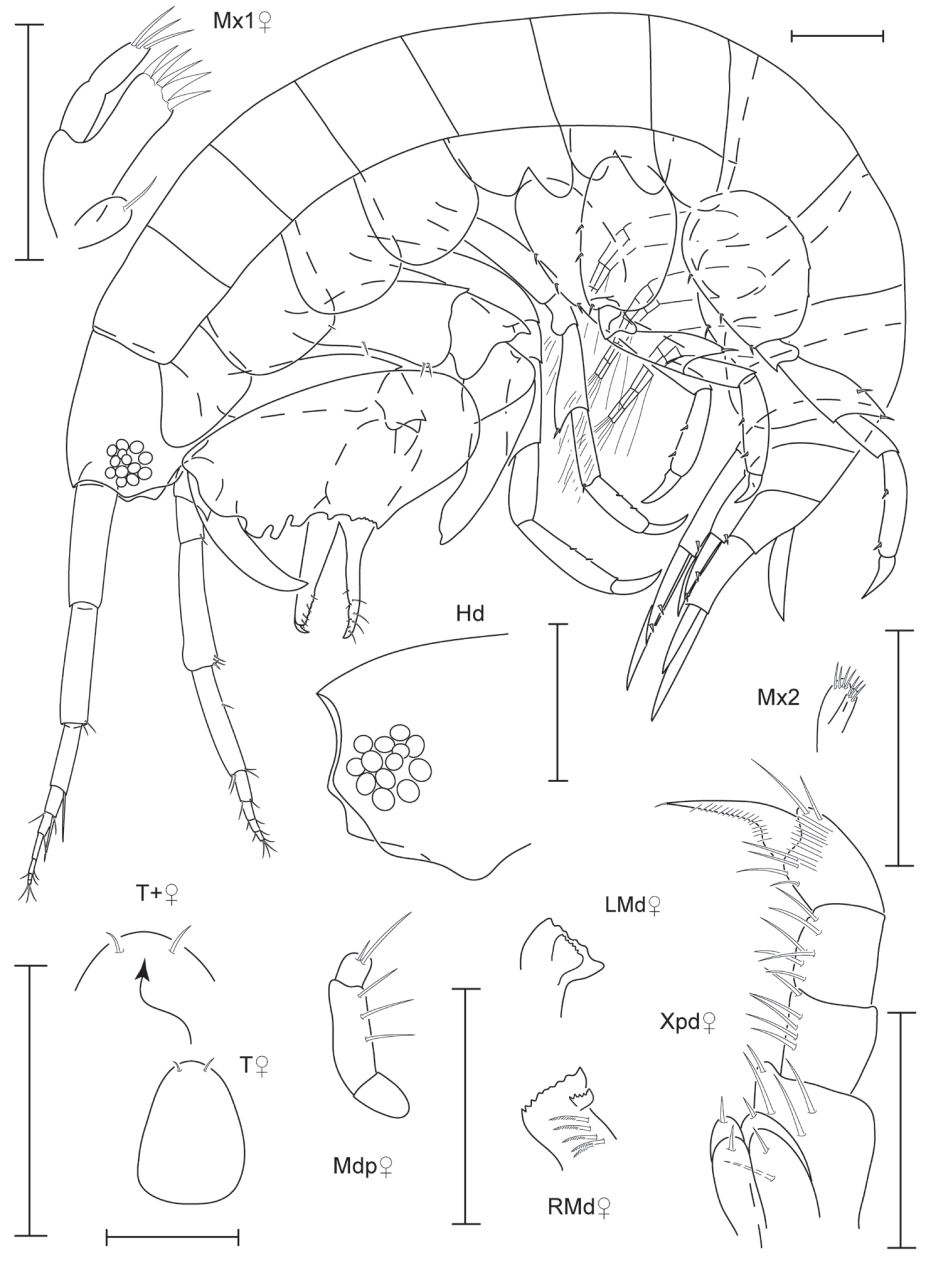
*Leucothoe enko* sp. n., holotype male, 1.6 mm, RUMF-ZC-1850; paratype female, 1.7 mm, RUMF-ZC-1851.

**Figure 10.**
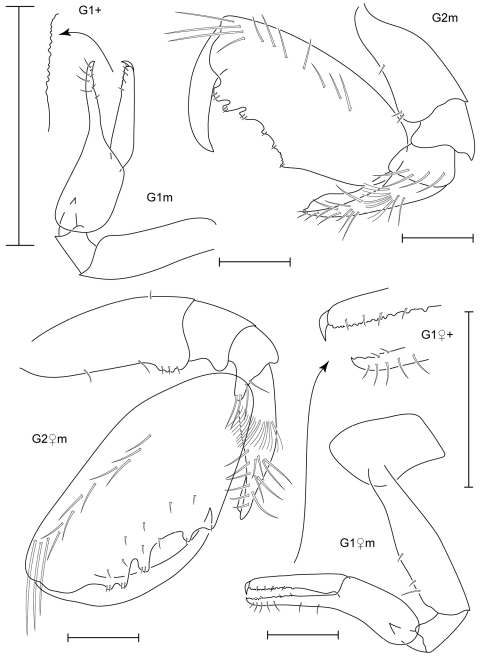
*Leucothoe enko* sp. n., holotype male, 1.6 mm, RUMF-ZC-1850; paratype female, 1.7 mm, RUMF-ZC-1851.

Pleon. Epimera 1–2 with ventral setae, epimeron 3 bare, posteroventral corner subquadrate. Uropods 1–3 relative lengths 1.0: 0.6: 0.9. Uropods 1–2 peduncles 0.8 × inner rami lengths, outer rami 0.6 × inner rami lengths. Uropod 1 inner ramus with 2 robust setae, outer ramus with 1 robust seta. Uropod 2 inner and outer rami each with 1 robust seta. Uropod 3 peduncle and inner ramus 0.7 × inner ramus length; inner and outer rami bare. Telson 1.8 × longer than wide, apex rounded.

#### Female (sexually dimorphic characters).

 Gnathopod 1 basis anterior margin with 3 short setae; carpus distal margin with 7 long setae. Gnathopod 2 basis anterior margin with 3 distal tubercules, with 3 short and 2 medium setae, posterior margin with 1 short seta; propodus with 2 rows of submarginal setae, palm 5 major tubercles; dactylus reaching 0.7 × propodus length.

#### Etymology.

 After the Japanese word ‘enko’, meaning ‘arc’ and referring to the concave gnathopod 2 propodus palm (pronounced en-koh).

#### Ecology.

Host unknown, presumably living in sponges in coral rubble.

#### Relationships.

*Leucothoe enko* sp. n. fits into a distinct group of small species sharing a proximally inflated gnathopod 1 propodus and very strong dentition on the propodus palm of gnathopod 2. These species include *Leucothoe adelphe* White & Thomas, 2009, *Leucothoe micronesiae* Barnard, 1965, and *Leucothoe minuscula* Schellenberg, 1938. *Leucothoe enko* sp. n. differs from these species in having the maxilla 1 palp 1–articulate with margins constricted, a proximally dentate gnathopod 1 carpus and propodus, posteriorly serrate pereopods 5–7 bases, and telson with a rounded apex.

#### Remarks.

*Leucothoe enko* sp. n. is deep yellow in color with a small red eye (Figure 15C). This species is endemic to the central Ryukyu Islands. Upon further examination, this group of species may prove to be a part of a cryptic species complex, with different species inhabiting similar niches at different locations.

#### Distribution.

 East China Sea: Okinawa–jima Island (Okinawa), Okinoerabu–jima Island and Tokunoshima Island (both Kagoshima), Japan.

### Leucothoe kebukai sp. n.

urn:lsid:zoobank.org:act:67D3C0E3-1116-4C18-BB11-BB8536620BD3

http://species-id.net/wiki/Leucothoe_kebukai

Figures 11 12

#### Type material.

Holotype male, 2.2 mm RUMF-ZC-1865, Yona, Kunigami, Okinawa–jima Island, Okinawa, reef wall (26°45'56"N, 128°11'46"E), among coral rubble, 9 m, K.N. White and N.S. White, col., 23 October 2010 (KNWOkinawa15H). Paratype female, 2.6 mm, RUMF-ZC-1866, Kaminomine, Tokunoshima Island, Kagoshima, patch reef (27°46'11"N, 129°02'19"E), among coral rubble, 1 m, K.N. White and N.S. White, col., 20 September 2010 (KNWTokuno1A).

#### Type locality.

 Yonna, Kunigami, Okinawa–jima Island, Okinawa, Japan (26°45'56"N, 128°11'46"E).

#### Additional material examined.

 1 specimen, NSMT-Cr 22002, KNWOkinawa14D; 1 specimen, RUMF-ZC-1867, KNWOkinawa15H; 3 specimens, NSMT-Cr 22003, KNWOkinawa15H; 1 specimen, NSMT-Cr 22004, KNWOkinawa19B;1 specimen, RUMF-ZC-1868, KNWOkinawa19C; 1 specimen, RUMF-ZC-1869, KNWOkinawa31C; 1 specimen, NSMT-Cr 22005, KNWOkinawa33F; 3 specimens, RUMF-ZC-1870, KNWOkinawa34L; 7 specimens, NSMT-Cr 22006, KNWOkinawa53E; 1 specimen, RUMF-ZC-1871, KNWOkinawa60A; 1 specimen, NSMT-Cr 22007, KNWTokuno3C; 3 specimens, RUMF-ZC-1872, KNWTokuno3E; 2 specimens, NSMT-Cr 22008, KNWTokuno4G; 1 specimen, RUMF-ZC-1873, KNWYaku1E; 1 specimen, NSMT-Cr 22009, KNWYaku2D.

#### Diagnosis (male).

 Head anterior margin truncate, distal margin oblique, convex. Right mandible lacinia mobilis represented by a small ridge. Maxilla 1 palp 1–articulate, margins constricted. Gnathopod 1 carpus and propodus curved; dactylus reaching 0.2 × propodus length. Gnathopod 2 basis anterior margin with 7 long setae; carpus distally truncate, spoon-like. Pereopods 5–6 coxae with facial setae; bases broadly expanded.

#### Description (male).

 Head. Anterior margin truncate, anterodistal margin subquadrate, distal margin oblique, convex; ventral cephalic keel anterior margin transverse, anteroventral margin subquadrate, ventral margin straight; eyes present with more than 10 ommatidia, round. Antenna 1 0.4 × body length, flagellum 7–articulate, peduncle article 1 width less than 2 × article 2, accessory flagellum absent. Antenna 2 0.3 × body length, shorter than antenna 1, flagellum 7–articulate. Mandibular palp ratio of articles 1–3 1.0: 3.0: 0.6, article 2 with 2–3 distal setae, article 3 with 1distal seta, incisors weakly dentate; left mandible with 9 raker spines, lacinia mobilis large, strongly toothed; right mandible with 7 raker spines, lacinia mobilis represented by a small ridge. Upper lip asymmetrically lobate, anterior margin setose. Lower lip inner lobes fused, bare; outer lobes with moderate gape, anterior margins setose. Maxilla 1 palp 1–articulate, margins constricted, with 3 distal slender setae; outer plate with 6 distal robust setae. Maxilla 2 inner plate with 3 slender distal, 3 marginal, and 1 facial seta; outer plate with 4 distal slender setae and 4 marginal slender setae. Maxilliped inner plates distal margin with v-shaped indentation, with short simple robust setae; outer plate inner margin smooth, reaching 0.1 × palp article 1, facial setae absent; palp article 4 subequal in length with article 3, distally acute.

Pereon. Coxae 1–4 relative widths 1.0: 1.1: 1.1: 1.2. Gnathopod 1 coxa smooth, with tiny marginal setae, anterodistal margin produced, rounded, distal margin rounded, posterior margin slightly excavate, facial setae absent; basis distally expanded, anterior margin with 1 medium seta, posterior margin bare; ischium bare; carpus curved, distal length 7.4 × width, proximal margin smooth, distal margin with 1 seta; propodus curved, palm smooth with 12 distal setae; dactylus smooth, reaching 0.2 × propodus length. Gnathopod 2 coxa longer than broad, slightly larger than coxa 3, smooth, with tiny marginal setae; anterior margin rounded, anterodistally rounded, distal margin rounded, posterior margin straight, facial setae absent; basis distally expanded, anterior margin with 7 long setae, posterior margin bare; ischium with 3 posterodistal setae; carpus 0.2 × propodus length, curved, distally truncate, spoon-like, anterior margin smooth; propodus with 1 mediofacial setal row above midline, reaching 0.6 × propodus length, with 1 row of submarginal setae, posterior margin smooth, palm convex, with 3 small tubercles; dactylus curved, proximal margin smooth, bare, anterior margin distally acute, reaching 0.5 × propodus length. Pereopod 3 coxa length 0.9 × width, anterodistal corner overriding distal face of coxa 2, not extending below it, smooth, with tiny marginal setae, anterior margin rounded, distal and posterior margins straight, facial setae absent. Pereopod 4 coxa smooth, with tiny marginal setae, anterior and distal margins rounded, posterior margin tapered, facial setae absent. Pereopods 5–6 coxae facial setae present, pereopod 7 coxa facial setae absent. Pereopods 5–7 bases width length ratios 1: 1.5, 1: 1.5, 1: 1.6, posterior margins smooth, setose.

Pleon. Epimera 1–2 with ventral setae, epimeron 3 bare, posteroventral corner subquadrate, produced. Uropods 1–2 relative lengths 1.0: 0.9. Uropod 1 peduncle 1.0 × inner ramus length, outer ramus 0.8 × inner ramus length; inner and outer rami each with 2 robust setae. Uropod 2 peduncle 1.1 × inner ramus length, outer ramus 0.9 × inner ramus length; inner ramus with 3 robust setae, outer ramus with 2 robust setae. Uropods 1–2 inner and outer rami with robust setae. Uropod 3 missing. Telson 1.8 × longer than wide, apex tridentate.

**Figure 11.**
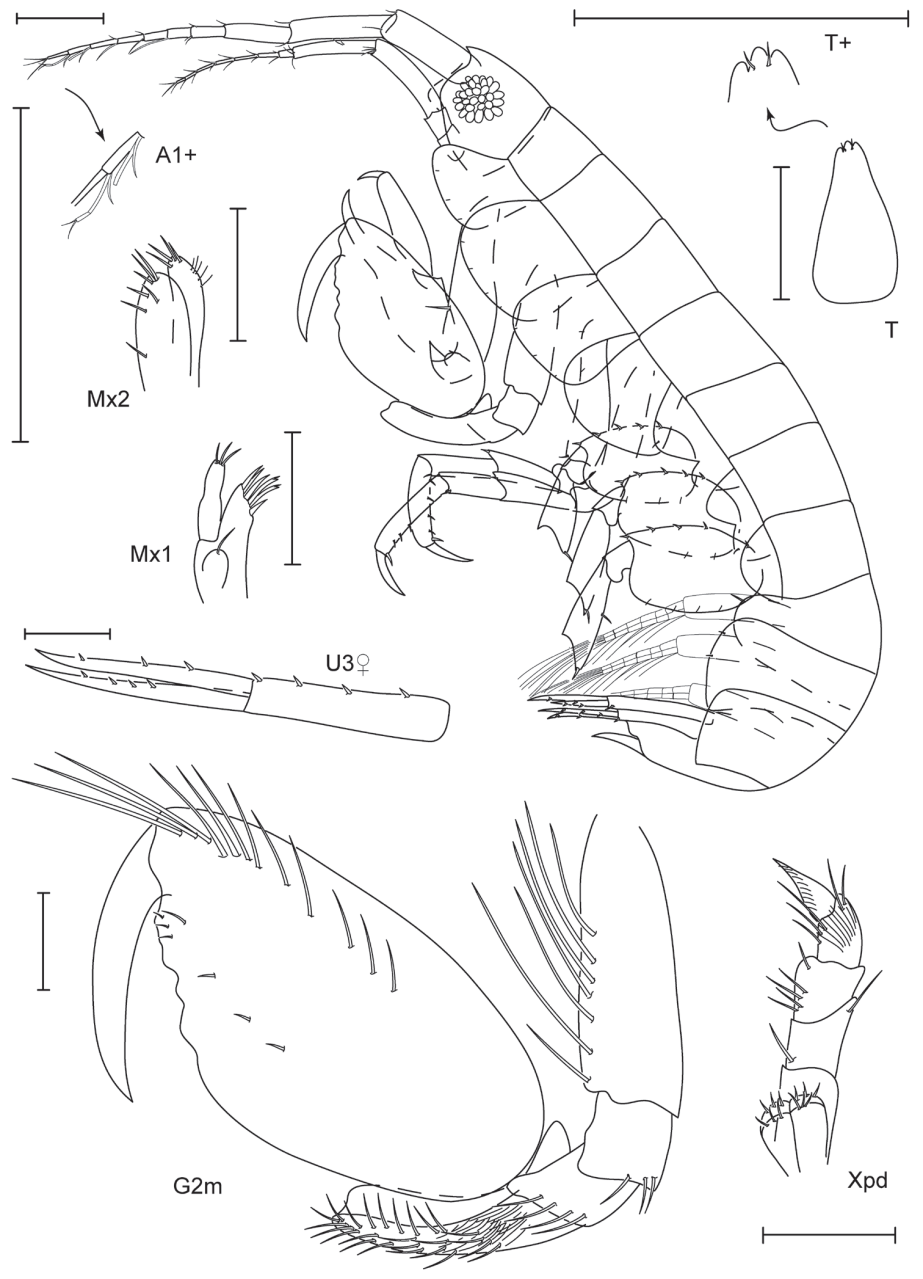
*Leucothoe kebukai* sp. n., holotype male, 2.2 mm, RUMF-ZC-1865; paratype female, 2.6 mm, RUMF-ZC-1866.

**Figure 12.**
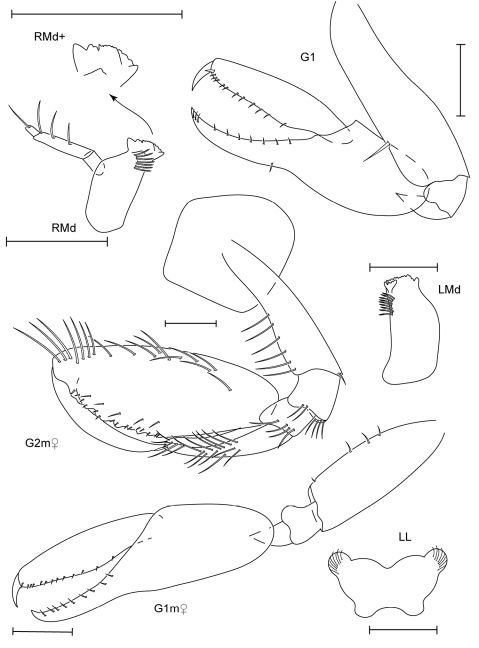
*Leucothoe kebukai* sp. n., holotype male, 2.2 mm, RUMF-ZC-1865; paratype female, 2.6 mm, RUMF-ZC-1866.

#### Female (sexually dimorphic characters).

 Gnathopod 1 basis distally expanded, anterior margin with 4 short setae; carpus distal margin bare; propodus palm with 13 distal setae. Gnathopod 2 basis anterior margin with 6 long setae, posterior margin with 1 posterodistal seta; ischium with 2 distal and 4 posterodistal setae; carpus distally tapered. Uropod 3 peduncle 0.9 × inner ramus length, outer ramus subequal in length with inner ramus; inner ramus with 4 robust setae, outer ramus with 3 robust setae.

#### Etymology.

 After the Japanese word ‘kebukai’, meaning ‘hairy’ and referring to the long setae on gnathopod 2 basis anterior margin (pronounced kay-boo-kigh).

#### Ecology.

 Host unknown, presumably in sponges in coral rubble.

#### Relationships.

*Leucothoe kebukai* sp. n. shares a distinctly curved and inflated gnathopod 1 propodus and a short gnathopod 1 dactylus with *Leucothoe alata* Barnard, 1959, *Leucothoe nagatai* Ishimaru, 1985, *Leucothoe obuchii* White & Reimer, 2012a, and *Leucothoe trulla*. *Leucothoe kebukai* sp. n. differs from all of these species in the following: maxilla 1 palp with an unclear articulation; gnathopod 2 basis anterior margin with seven long setae, ischium with posterodistal setae, propodus slender, and dactylus short.

#### Remarks.

*Leucothoe kebukai* sp. n. is translucent yellow in color with faint pink stripes along pereonite edges (Figure 15E). This species is endemic to the central and northern Ryukyu Islands.

#### Distribution.

 East China Sea: Okinawa–jima Island (Okinawa), Tokunoshima Island, Yakushima Island (both Kagoshima), Japan.

### Paranamixis

Schellenberg, 1938

http://species-id.net/wiki/Paranamixis

Figures 13 , 14

Paranamixis misakiensis Thomas, 1997: 89–91, fig. 26.

#### Material examined.

Anamorph male, 3.5 mm RUMF-ZC-1875, Haruta, Yakushima Island, Kagoshima, reef wall (30°18'12"N, 130°39'28"E), among coral rubble, 10 m, K.N. White and N.S. White, col., 27 May 2011 (KNWYaku4C).

#### Diagnosis (Anamorph male).

 Head anterior margin oblique, anterodistal margin oblique with notch; ventral cephalic keel anterior margin oblique with projection and excavation. Antenna 2 longer than antenna 1. Maxilliped inner plates fused with apical notch. Gnathopod 1 coxa anterodistal margin subtriangular, bi-cuspidate. Gnathopod 2 coxa broader than long, greatly enlarged, distal margin with anterior and posterior cusps; basis anterior margin with distal serrate ridge; carpus distally tapered, anterior margin dentate; dactylus proximal margin dentate with 2 plumose setae. Telson apex rounded.

#### Description (Anamorph male).

 Head. Anterior margin oblique, anterodistal margin oblique with notch, distal margin rounded; ventral cephalic keel anterior margin oblique with projection and excavation, anteroventral margin quadrate, produced, ventral margin oblique; eyes present with more than 10 ommatidia, round. Antenna 1 0.3 × body length, flagellum 7–articulate, peduncle article 1 width less than 2 × article 2, accessory flagellum 1–articulate. Antenna 2 0.4 × body length, longer than antenna 1, flagellum 3–articulate. Mouthparts reduced. Maxilliped inner plates fused with apical notch; outer plate inner margin smooth, reaching 0.1 × palp article 1, bare; palp 4–articulate, article 4 elongate, distally acute.

Pereon. Coxae 1–4 relative widths 1.0: 2.9: 1.7: 2.0. Gnathopod 1 absent, coxa smooth, with tiny marginal setae, anterodistal margin produced, subtriangular, bi-cuspidate, distal margin oblique, posterior margin straight, facial setae absent. Gnathopod 2 coxa broader than long, greatly enlarged, smooth, bare, anterior margin rounded, anterodistally rounded, distal margin rounded with anterior and posterior cusps, posterior margin rounded, facial setae absent; basis distally expanded, anterior margin with distal serrate ridge and 7 short setae, posterior margin with 1 posterodistal seta; ischium bare; carpus 0.8 × propodus length, curved, distally tapered, anterior margin dentate; propodus with 1 mediofacial setal row above midline, reaching 0.5 × propodus length, with 1 row of submarginal setae, posterior margin serrate, palm convex with 3 major and many small tubercles; dactylus curved, proximal margin dentate with 2 plumose setae, anterior margin distally obtuse, reaching 0.3 × propodus length. Pereopod 3 coxa length 1.4 × width, anterodistal corner overriding distal face of coxa 2, not extending below it, smooth, bare, anterior margin straight, distal margin straight with mid-distal cusp, posterior margin straight, facial setae absent. Pereopod 4 coxa smooth, bare, anterior margin straight, distal margin rounded with anterior and mid-distal cusps, posterior margin excavate, facial setae absent. Pereopods 5–7 coxae facial setae absent; bases width length ratios 1: 1.3, 1: 1.3, 1: 1.4, posterior margins smooth, setose.

Pleon. Epimera 1–3 bare; epimeron 3 posteroventral corner quadrate. Uropods 1–3 relative lengths 1.0: 0.8: 0.9. Uropod 1 peduncle 0.9 × inner ramus length, outer ramus 0.5 × inner ramus length; inner ramus with 4 robust setae, outer ramus with 2 robust setae. Uropod 2 peduncle 0.8 × inner ramus length, outer ramus 0.6 × inner ramus length; inner and outer rami each with 3 robust setae. Uropod 3 peduncle 1.3 × inner ramus length, outer ramus 0.7 × inner ramus length; inner ramus bare, outer ramus with one robust seta. Telson 1.1 × longer than wide, apex rounded.

**Figure 13.**
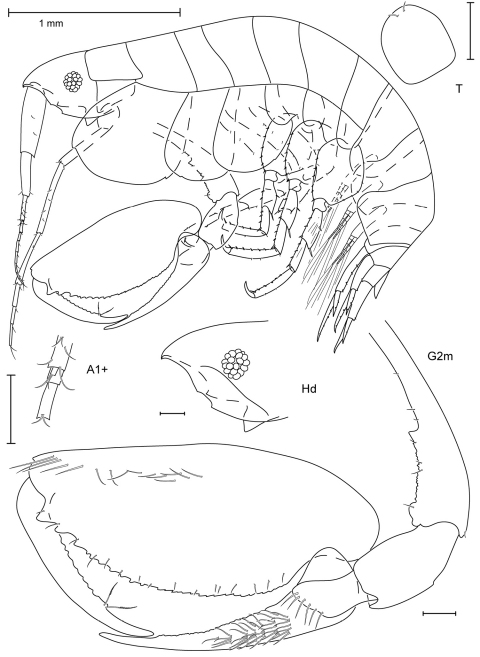
*Paranamixis misakiensis* Thomas, 1997, male anamorph, 3.5 mm, RUMF-ZC-1875.

**Figure 14.**
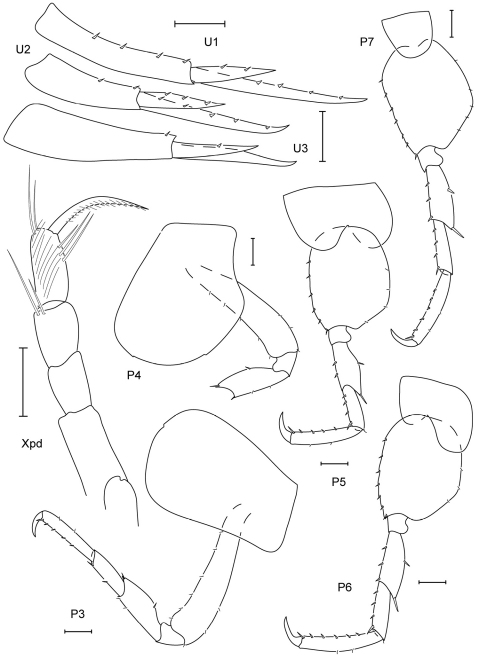
*Paranamixis misakiensis* Thomas, 1997, male anamorph, 3.5 mm, RUMF-ZC-1875.

#### Leucomorph (juvenile and sexually dimorphic characters).

Unknown.

#### Ecology.

 Host unknown, presumably in sponges in coral rubble.

#### Relationships.

*Paranamixis misakiensis* appears most closely related to *Paranamixis thomasi* in the cuspate, oblique anterodistal head margin, excavate anterior ventral cephalic keel margin, apically cleft maxilliped inner plates, and enlarged gnathopod 2 coxa. *Paranamixis misakiensis* differs from *Paranamixis thomasi* in the following: ventral cephalic keel anterior margin with mid-distal projection; gnathopod 2 basis anterior margin with a much larger serrate ridge, propodus with one mediofacial setal row, dactylus proximal margin and dentate. *Paranamixis denticulus* Kim and Kim, 1991 and *Paranamixis aberro* Hirayama 1983 also share the oblique, cuspate anterior head margin, but differ in many other aspects.

#### Remarks.

**Figure 15.**
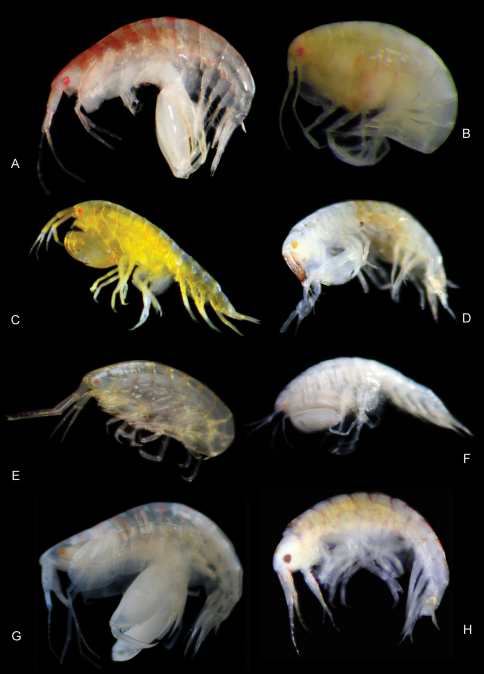
Color plate of new leucothoid amphipod species. **A** *Anamixis sentan* sp. n. anamorph male **B** *Anamixis sentan* sp. n. leucomorph female **C** *Leucothoe enko* sp. n. **D** *Leucothoe akaisen* sp. n. **E** *Leucothoe kebukai* sp. n. **F** *Leucothoe chiisainame* sp. n. **G** *Paranamixis misakiensis* Thomas, 1997 **H** *Leucothoe akuma* sp. n.

 Anamorph males of *Paranamixis misakiensis* is white in color with pink stripes along pereonite edges (Figure 15G). This species was collected from Yakushima Island, extending its known range approximately 1000 kilometers. The specimen from Yakushima closely agrees with Thomas’ 1997 description, differing slightly in the ventral cephalic keel, which is excavate with a mid-distal projection, versus excavate in Thomas’ 1997 material.

**Figure 16.**
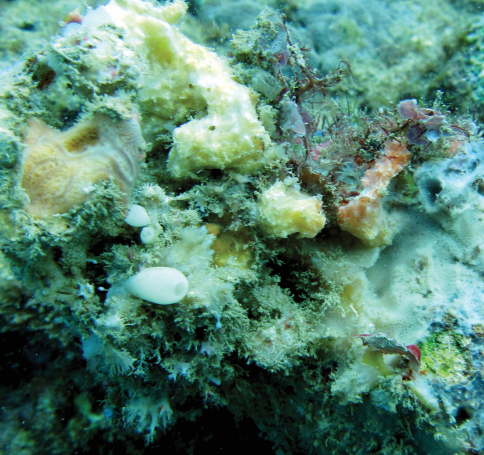
Sponge-filled coral rubble.

#### Distribution.

 East China Sea: Yakushima Island (Kagoshima), Japan. Pacific Ocean: Misaki, Miura Peninsula (Kanagawa), Japan.

### Identification Key for all Leucothoidae of the Ryukyu Archipelago

#### (characters refer to males unless noted)

**Table:** 

1	Extreme sexual dimorphism; coxa 1 reduced; mouthparts reduced in adult males	2
–	Minimal sexual dimorphism; coxa 1–4 relatively equal in widths; mouthparts well developed	4
2	Anamorph head anterior margin truncate; gnathopod 1 present in post-transformational molts; gnathopod 2 coxa longer than broad, slightly enlarged. Leucomorph gnathopod 1 coxa anterodistal margin subtriangular, propodus straight	*Anamixis sentan* sp. n.
–	Anamorph head anterior margin oblique; gnathopod 1 absent in post-transformational molts; gnathopod 2 coxa broader than long, greatly enlarged. Leucomorph gnathopod 1 coxa anterodistal margin rounded, propodus curved	3
3	Anamorph ventral cephalic keel ventral margin subquadrate; antenna 1 accessory flagellum absent; gnathopod 2 coxa anteriorly expanded, basis anterior margin with small serrate ridge, dactylus proximal margin with 1 tubercle	*Paranamixis thomasi*
–	Anamorph ventral cephalic keel ventral margin oblique; antenna 1 accessory flagellum 1–articulate; gnathopod 2 coxa rounded, basis anterior margin with pronounced serrate ridge, dactylus proximal margin dentate	*Paranamixis misakiensis*
4	Gnathopod 1 dactylus reaching no more than 0.2 × propodus length	5
–	Gnathopod 1 dactylus reaching more than 0.2 × propodus length	15
5	Gnathopod 1 carpus basally inflated; gnathopod 2 propodus mediofacial setal row very robust with tufts of setae, palm with large indentation	*Leucothoe nathani*
–	Gnathopod 1 carpus linear; gnathopod 2 propodus mediofacial setal row(s) without tufts of setae, palm without large indentation	6
6	Gnathopod 1 propodus proximally inflated; gnathopod 2 propodus palm concave; telson apex rounded	*Leucothoe enko* sp. n.
–	Gnathopod 1 propodus straight or curved; gnathopod 2 propodus palm convex; telson apex tridentate, bidentate, or truncate	7
7	Antenna 1 reaching greater than 0.5 × body length, peduncle article 1 width greater than 2 × article 2; eyes rectangular; gnathopod 1 coxa about ½ as wide as coxa 2; telson apex truncate	*Leucothoe zanpa*
–	Antenna 1 reaching less than 0.5 × body length, peduncle article 1 width less than 2 × article 2; eyes round or oval; gnathopod 1 coxa more than ½ as wide as coxa 2; telson apex tridentate or bidentate	8
8	Eye large, covering most head; gnathopod 1 elongate; gnathopod 2 propodus with one submarginal row of robust setae; female gnathopod 1 basis posterior margin with 15 medium setae	*Leucothoe elegans*
–	Eye small or moderate in size; gnathopod 1 not elongate; gnathopod 2 propodus without robust submarginal setae; female gnathopod 1 basis posterior margin bare or with less than 15 setae	9
9	Gnathopod 1 propodus curved, inflated, carpus robust	10
–	Gnathopod 1 propodus straight, carpus slender	12
10	Head anterior margin rounded, antenna 2 flagellum 3–articulate; maxilliped outer plate inner margin reaching greater than 0.5 × palp article 1; gnathopod 1 carpus proximal margin dentate; gnathopod 2 carpus distally tapered, propodus with mediofacial setal row displaced to midline	*Leucothoe obuchii*
–	Head anterior margin truncate, antenna 2 flagellum 6+–articulate; maxilliped outer plate inner margin reaching less than 0.5 × palp article 1; gnathopod 1 carpus proximal margin smooth; gnathopod 2 carpus distally truncate, spoon-like, propodus with primary mediofacial setal row above midline	11
11	Head anterodistal margin subquadrate; antenna 1 accessory flagellum absent; gnathopods 1 and 2 bases posterior margins bare; gnathopod 2 propodus with 1 mediofacial setal row; pereopods 5–7 bases narrowly expanded	*Leucothoe kebukai* sp.n.
–	Head anterodistal margin concave; antenna 1 accessory flagellum 1–articulate; gnathopods 1 and 2 bases posterior margins setose; gnathopod 2 propodus with 2 mediofacial setal rows; pereopods 5–7 bases broadly expanded	*Leucothoe toribe*
12	Antenna 1 accessory flagellum absent; gnathopod 1 carpus proximal margin smooth, propodus palm without large triangular teeth; telson bidentate	*Leucothoe ouraensis*
–	Antenna 1 accessory flagellum 1–articulate; gnathopod 1 carpus proximal margin serrate or with denticles, propodus palm with large triangular teeth; telson tridentate	13
13	Head anterior margin truncate, anterodistal margin quadrate; gnathopod 1 basis posterior margin bare; gnathopod 2 propodus with 1 mediofacial setal row	*Leucothoe nagetekubi*
–	Head anterior and anterodistal margins rounded; gnathopod 1 basis posterior margin setose; gnathopod 2 propodus with 2 mediofacial setal rows	14
14	Ventral cephalic keel anteroventral margin subquadrate; eye round; antenna 1 flagellum 5–articulate; gnathopod 1 carpus and propodus extremely slender in appearance; pereopods 5–7 bases narrowly expanded, posteriorly smooth	*Leucothoe hashi*
–	Ventral cephalic keel anteroventral margin quadrate with small projection; eye oval; antenna 1 flagellum 10+–articulate; gnathopod 1 carpus and moderately slender in appearance; pereopods 5–7 bases broadly expanded, posteriorly serrate	*Leucothoe lecroyae*
15	Head anterior margin truncate, anterodistal margin quadrate; gnathopod 2 propodus with 2 mediofacial setal rows, secondary row with 1–4 setae near insertion of carpus; pereopods 5–7 bases narrowly expanded	*Leucothoe bise*
–	Head anterior margin rounded or excavate, anterodistal margin rounded or subquadrate; gnathopod 2 propodus with 1 or 2 mediofacial setal row, secondary row, if present, with 6 setae reaching to insertion of dactylus; pereopods 5–7 bases broadly expanded	16
16	Coxae 1–4 with lateral facial setae; gnathopod 2 carpus with sub-distal tooth; telson apex truncate; telson with simple marginal and plumose facial setae	*Leucothoe amamiensis*
–	Coxae 1–4 without lateral facial setae; gnathopod 2 carpus distally tapered or truncate; telson apex tridentate, rounded, or with strongly rounded point; telson without marginal setae, with or without simple facial setae	17
17	Ventral cephalic keel anteroventral margin quadrate, projected anteriorly, without cusp; gnathopod 2 propodus primary mediofacial setal row above midline	18
–	Ventral cephalic keel anteroventral margin quadrate, with cusp, rounded, or subquadrate; gnathopod 2 propodus primary mediofacial setal row displaced to or past midline	19
18	Gnathopod 2 basis anterior margin with several short and long setae, carpus distally truncate, anterior margin dentate, propodus with 2 mediofacial setal rows and 1 row of submarginal setae	*Leucothoe akuma* sp. n.
–	Gnathopod 2 basis anterior margin with several long curved setae, carpus distally tapered, anterior margin smooth, propodus with 1 mediofacial setal row, with 3 rows of submarginal setae	*Leucothoe togatta*
19	Coxa 1 posterior margin serrate; gnathopod 2 carpus distally truncate, spoon-like; pereopods 5–7 bases posterior margins bare	*Leucothoe trulla*
–	Coxa 1 posterior margin smooth; gnathopod 2 carpus distally tapered or truncate, if truncate then not spoon-like; pereopods 5–7 bases posterior margins setose	20
20	Ventral cephalic keel anteroventral margin with anteriorly projecting cusp; female gnathopod 2 basis anterior margin with ~30 setae; pereopods 5–7 bases oval	*Leucothoe akaoni*
–	Ventral cephalic keel anteroventral margin rounded or subquadrate; female gnathopod 2 basis anterior margin with less than 20 setae; pereopods 5–7 bases rounded or posteriorly tapered	21
21	Head anterior margin excavate; ventral cephalic keel ventral margin convex; eye very small; antenna 1 with 1–articulate accessory flagellum; gnathopod 2 dactylus reaching less than 0.5 × propodus length; pereopods 5–7 bases posteriorly serrate	*Leucothoe chiisainame* sp. n.
–	Head anterior margin rounded; ventral cephalic keel ventral margin straight or oblique; eye medium or large; antenna 1 lacking accessory flagellum; gnathopod 2 dactylus reaching greater than 0.5 × propodus length; pereopods 5–7 bases posteriorly smooth	22
22	Head anterodistal margin subquadrate; coxa 1 anterodistal corner rounded, lacking medial facial setae; gnathopod 2 basis posterior margin setose; coxa 4 posterior margin tapered; epimeron 1 bare; telson apex tridentate	*Leucothoe daisukei*
–	Head anterodistal margin rounded; coxa 1 anterodistal corner subquadrate, with 1 facial seta; gnathopod 2 basis posterior margin bare; coxa 4 posterior margin excavate; epimeron 1 setose; telson apex rounded or with a strongly rounded point	23
23	Ventral cephalic keel anterior margin transverse; antenna 1 flagellum 5–articulate; gnathopod 2 carpus distally tapered, telson apex rounded	*Leucothoe akaisen*sp. n.
–	Ventral cephalic keel anterior margin excavate; antenna 1 flagellum 9–11–articulate; gnathopod 2 carpus distally truncate, telson apex with strongly rounded point	24
24	Antenna 2 flagellum 4–articulate; maxilla 1 palp 2–articulate; gnathopod 1 coxa anterior margin serrate, basis posterior margin bare, carpus distal margin setose, proximal margin dentate; gnathopod 2 basis posterior margin bare, carpus anterior margin dentate; epimeron 1 with tuft of anteroventral setae	*Leucothoe vulgaris*
–	Antenna 2 flagellum 7–articulate; Maxilla 1 palp 1–articulate, margins constricted; gnathopod 1 coxa anterior margin smooth, basis posterior margin setose, carpus distal margin bare, proximal margin smooth; gnathopod 2 basis posterior margin setose, carpus anterior margin smooth; epimeron 1 with ventral setae	*Leucothoe nurunuru*

## Discussion

*Leucothoe akaisen* sp. n. and *Leucothoe chiisainame* sp. n. share a displaced mediofacial setal row, which is typically found in ascidian-dwelling species worldwide, suggesting that these species may also inhabit ascidian hosts, or that this character may be homologous among *Leucothoe* species and not an artifact of convergent evolution, as noted in White and Reimer (2012a). Three species described here have a small accessory flagellum on antenna 1 (*Leucothoe akuma* sp. n., *Leucothoe chiisainame* sp. n., and *Paranamixis misakiensis*). This character is unusual among leucothoid species, but apparently is much more common in Pacific species than in Caribbean species.

A range extension is reported for *Paranamixis misakiensis* which was previously known from only Misaki, Kanagawa prefecture, Japan. Both Thomas’ (1997) report and this research have found this species from coral rubble, without more specific host data. Leucomorphs are currently unknown for this species, highlighting the need for further investigation into connecting life stages for the anamixid clade. Both *Paranamixis thomasi* and *Anamixis sentan* sp. n. anamorphs are connected to their leucomorph counterparts due to diligent collecting and recording of important host data. However, in most cases this specialized collecting is not undertaken, leaving morphologically different life stages unknown.

*Leucothoe chiisainame* sp. n. is reported from only one island, while all other new species here have been collected from at least two islands. *Leucothoe akaisen* sp. n. has been collected from throughout the entire Ryukyu Archipelago, *Leucothoe kebukai* sp. n. has been collected from the central and northern islands, *Anamixis sentan* sp. n. has been collected from the southern and central islands, and all other species described here are reported from only the central islands.

The currently recognized biogeographic boundaries of the Ryukyu Archipelago (Kizaki and Oshiro 1977, 1980; Hikida and Ota 1997; Ota 1998; Ota et al. 2004) do not appear to apply to all leucothoid amphipods in this region. Of the 25 leucothoid species collected in the Ryukyu Archipelago, two species were collected from only above the Watase Line at the Tokara Strait, two species were collected from only below the Hachisuka Line at the Kerama Gap, and eight were collected within these two boundaries (see map of boundaries in White and Reimer 2012a). The other 14 species ranges cross these boundaries. It is possible that further collections will reveal that the boundaries do not apply to any of these species or that host specialization limits the ranges of certain species and not of others.

The very high diversity of new leucothoid species discovered in the Ryukyu Archipelago to date supports the observation of Roberts et al. (2002), stating that Indo-Pacific reefs and in particular southern Japan and Taiwan include some of the most diverse areas in the world with high levels of endemicity. Taking this into account, this research noticed a higher species richness among leucothoids when comparing with a recent survey done in the Great Barrier Reef. This study reports 25 leucothoid species in 3 genera from 108 samples at 47 locations spanning approximately 1000 kilometers compared with 17 leucothoid species in 3 genera from 225 samples at 43 locations spanning approximately 1100 kilometers at Lizard Island, Australia and including samples from Orpheus and Heron Islands. Perhaps the incredible Leucothoidae diversity found in the Ryukyu Archipelago will apply to other amphipod families. It is clear that further sampling and research is needed in this region for a better understanding of the Amphipoda diversity.

## Supplementary Material

XML Treatment for Anamixis

XML Treatment for Anamixis sentan

XML Treatment for Leucothoe

XML Treatment for Leucothoe akaisen

XML Treatment for Leucothoe akuma

XML Treatment for Leucothoe chiisainame

XML Treatment for Leucothoe enko

XML Treatment for Leucothoe kebukai

XML Treatment for Paranamixis
